# N‐Doped Carbon Nanotubes Derived from Graphene Oxide with Embedment of FeCo Nanoparticles as Bifunctional Air Electrode for Rechargeable Liquid and Flexible All‐Solid‐State Zinc–Air Batteries

**DOI:** 10.1002/advs.202004572

**Published:** 2021-03-16

**Authors:** Xiaoqiong Hao, Zhongqing Jiang, Baoan Zhang, Xiaoning Tian, Changsheng Song, Likui Wang, Thandavarayan Maiyalagan, Xiaogang Hao, Zhong‐Jie Jiang

**Affiliations:** ^1^ Key Laboratory of Optical Field Manipulation of Zhejiang Province Department of Physics Zhejiang Sci‐Tech University Hangzhou 310018 P. R. China; ^2^ Department of Chemical Engineering Taiyuan University of Technology Taiyuan 030024 P. R. China; ^3^ Department of Materials and Chemical Engineering Ningbo University of Technology Ningbo 315211 P. R. China; ^4^ The Key Laboratory of Synthetic and Biological Colloids Ministry of Education School of Chemical and Materials Engineering Jiangnan University Wuxi 214122 P. R. China; ^5^ Electrochemical Energy Laboratory Department of Chemistry SRM Institute of Science and Technology SRM Nagar Kattankulathur 603203 India; ^6^ Guangdong Engineering and Technology Research Center for Surface Chemistry of Energy Materials and Guangzhou Key Laboratory for Surface Chemistry of Energy Materials New Energy Research Institute College of Environment and Energy South China University of Technology Guangzhou 510006 P. R. China

**Keywords:** all‐solid‐state Zn–air battery, bifunctional electrocatalyst, FeCo alloy, N,P codoped carbon, nitrogen‐doped carbon nanotube, rechargeable Zn–air battery

## Abstract

This work reports a novel approach for the synthesis of FeCo alloy nanoparticles (NPs) embedded in the N,P‐codoped carbon coated nitrogen‐doped carbon nanotubes (NPC/FeCo@NCNTs). Specifically, the synthesis of NCNT is achieved by the calcination of graphene oxide‐coated polystyrene spheres with Fe^3+^, Co^2+^ and melamine adsorbed, during which graphene oxide is transformed into carbon nanotubes and simultaneously nitrogen is doped into the graphitic structure. The NPC/FeCo@NCNT is demonstrated to be an efficient and durable bifunctional catalyst for oxygen evolution (OER) and oxygen reduction reaction (ORR). It only needs an overpotential of 339.5 mV to deliver 10 mA cm^−2^ for OER and an onset potential of 0.92 V to drive ORR. Its bifunctional catalytic activities outperform those of the composite catalyst Pt/C + RuO_2_ and most bifunctional catalysts reported. The experimental results and density functional theory calculations have demonstrated that the interplay between FeCo NPs and NCNT and the presence of N,P‐codoped carbon structure play important roles in increasing the catalytic activities of the NPC/FeCo@NCNT. More impressively, the NPC/FeCo@NCNT can be used as the air‐electrode catalyst, improving the performance of rechargeable liquid and flexible all‐solid‐state zinc–air batteries.

## Graphical abstract

The FeCo alloy nanoparticles embedded in the N,P‐codoped carbon coated nitrogen‐doped carbon nanotubes (NPC/FeCo@NCNT) have been synthesized and demonstrated to be efficient and durable catalysts for oxygen evolution and reduction reactions. It is usable as the air‐electrode catalysts to improve the performance of rechargeable liquid and flexible all‐solid‐state zinc–air batteries.

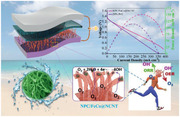

## 1 Introduction

Due to increasing environmental problems and rapid growth of global energy demand, search for novel and alternative energy conversion and storage devices has drawn global attention. Rechargeable zinc–air batteries (ZABs) have been considered as the promising candidates attracting tremendous attentions, because of their high theoretical energy density (1084 Wh kg^−1^), low cost of Zn metal (≈0.9 USD lb^−1^), environmental friendliness, and safety characteristics.^[^ ^1^ ^]^ However, the sluggish reaction kinetics of oxygen reduction reaction (ORR) and oxygen evolution reaction (OER) occurred at the anode of the ZABs have imposed a big constraint for their widespread applications. To date, the first‐ranked OER and ORR catalysts are noble metal‐based materials, such as Ir/C and RuO_2_ (for OER), and Pt (for ORR).^[^ ^2^ ^]^ These commercial catalysts, however, are confronted with the troubles that cannot simultaneously drive the OER and the ORR with high efficiency. This, along with the high price and poor durability of noble metal‐based materials, necessitates the development of the earth‐abundant, low‐cost, and high efficiency bifunctional catalyst for the ZABs.

During the past decades, tremendous efforts have been devoted to exploring nonprecious metal based bifunctional electrocatalysts, including composites which are composed of components with individual OER (e.g., transition metals, metallic alloys, metal oxides/phosphide, etc.) and ORR (such as carbon nanotubes (CNTs), graphene, activated carbon, heteroatom‐doped nanocarbon, etc.) catalytic activities. Among various methods reported, confining alloy nanoparticles (NPs) into carbon nanotubes have been demonstrated as a judicious choice,^[^ ^3^ ^]^ since such a structure is expectable to exhibit the following aspects of advantages: i) the alloy NPs deliver better activity than their individual components; ii) the encased alloy NPs sheathed by the carbon layer avoids the dissolution and erosion of metals by electrolyte and simultaneously prevents the aggregation of NPs; iii) the random stacking of the CNTs gives the high porosity to the catalyst, allowing its better accessibility to the catalytic reaction, and meanwhile, the sp^2^ hydrized structure of the CNTs improves the electrical conductivity of the catalyst, allowing a fast charge transfer during the catalytic reactions; iv) the heterogeneous interfaces and strong coupling between the alloy NPs and carbon shell is helpful to drive a fast reaction kinetics.

Up to now, a variety of alloy@carbon nanotube composite catalysts have been successfully synthesized by using different carbon and metal precursors and synthetic strategies. For instance, Wang et al. prepared FeNi alloy NPs embedded N‐doped CNTs‐tangled porous carbon fiber (FeNi/NCPCF) via the electrospinning and the subsequent annealing treatment.^[^ ^3^ ^]^ Wu et al. prepared FeNi NPs confined in N‐doped carbon by integrating metal–organic framework (MOF) precursor with polymer coating/encapsulation strategy.^[^ ^4^ ^]^ Gupta et al. reported FeCoNi ternary metal‐derived nitrogen‐doped graphene tube catalyst (N‐GT(FeCoNi).^[^ ^5^ ^]^ Wang et al. designed pod‐like N‐doped CNTs encapsulating FeCo alloy (FeCo/CNTs) catalyst by annealing the pure precursor Co_2_Fe(CN)_6_.^[^ ^6^ ^]^

Additionally, to further improve the catalytic activity of alloy@CNTs, several surface‐nanoengineering strategies have been proposed, including surface‐doping, surface‐coating, and their combination. Surface‐coating is a straightforward means useful for the regulation of the functionality and performance of catalysts because the coating skin can directly protect electrode catalysts against electrolyte.^[^ ^7^ ^]^ Carbon coating has been considered as one of the most frequently used and effective surface‐coating methods because its graphitic structure can readily enhance the electronic conductivity of catalysts and stabilize NPs incorporated as well. In particular, the doping heteroatoms, such as N and P, can provide an additional versatility for modification of surface electronic structures of carbon, optimizing the oxygen adsorption energy, reducing the kinetic energy barriers, and improving the intrinsic activity of catalytically active sites. The element N has higher electronegativity (3.04) than C (2.55). The doping of N can make the neighboring C atoms positively charged due to the electron transfer from C to N. The positively charged carbon atoms can then act as the active sites facilitating the adsorption of oxygen and the subsequent oxygen reduction. Furthermore, the similarity in the atomic radius between N and C is helpful to form the strong N—C covalent bond, increasing the durability of N—C active sites.^[^ ^8^ ^]^ The doping of P can rich edge‐defect sites on carbon and improve the catalytic activity of carbon as well. Although P has larger atomic radius and lower electronegativity (2.19) than those of C and favors the sp^3^‐orbital configuration, its doping in carbon can produce positively charged P^+^ sites. These charged P^+^ sites can also work as the active sites for the catalytic reactions.^[^ ^8^, ^9^ ^]^ It has been recently demonstrated that binary heteroatom codoping could generate synergistic coupling effects between the carbon frameworks and two kinds of heteroatoms, further enhancing the electrocatalytic activities of the catalysts for OER and ORR.^[^ ^10^ ^]^ For example, Chen et al. prepared N,P codoped 3D hierarchical carbons (NPHCs) ORR catalyst by using 4,4′‐bipyridine and phytic acid as N and P resources, respectively.^[^ ^11^ ^]^ Chen and co‐workers reported Fe_2_P/NPC ORR catalyst by utilizing d‐glucosamine hydrochloride and phytic acid as N and P resources, respectively.^[^ ^12^ ^]^ Niu et al. designed CoFe@NP‐CHS ORR/OER catalyst by adopting melamine and VB_12_ as N and P resources, respectively.^[^ ^13^ ^]^ Inspired by these findings, we conjecture that designing N‐doped CNTs (NCNTs) embedded with alloys with simultaneous coating of the N,P codoped carbon layer could provide bigger space for performance optimization of the alloy@CNT bifunctional electrocatalysts. Nevertheless, few successful productions of these materials with such configurations have been reported.

**Scheme 1:**
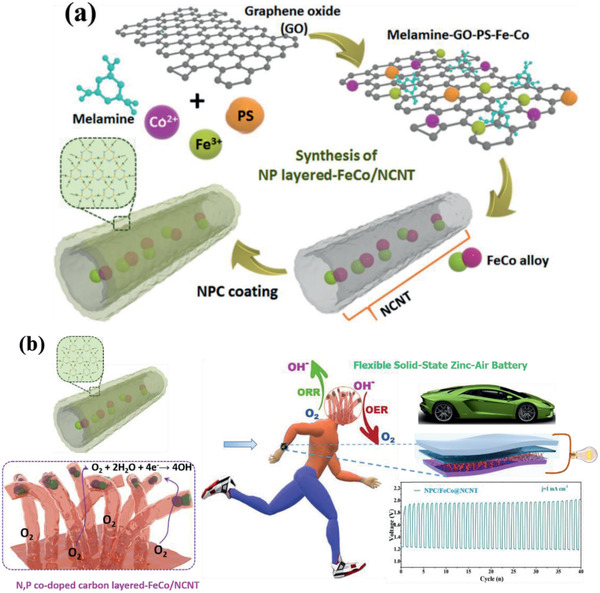
a) Schematic illustration of preparation of NPC/FeCo@NCNT, and b) its application in flexible wearable zinc–air batteries.

In this work, we report a novel approach for the production of FeCo alloy nanoparticles (NPs) embedded in N,P codoped carbon coated N‐doped carbon nanotubes (NPC/FeCo@NCNTs), in which the NCNTs is derived from the graphene oxide (GO) nanosheets and melamine. Specifically, as shown in **Scheme** **1**, the melamine–GO–PS–Fe–Co composite, obtained via strong complex interactions (such as, electrostatic interaction, covalent bond, and coordination bond) between melamine, GO, PS (polystyrene spheres), FeCl_3_, and Co(NO_3_)_2_,^[^ ^14^ ^]^ is used as the starting material. A two‐step calcination of the melamine–GO–PS–Fe–Co composite produces the N‐doped carbon nanotubes with FeCo NPs embedded (FeCo@NCNT). The formation of NPC/FeCo@NCNT is achieved by the calcination of the FeCo@NCNT in the presence of polyphosphazenes (Figure S1, Supporting Information). Interestingly, the NPC/FeCo@NCNT displays greatly improved electrocatalytic performance, when used as a bifunctional catalyst for ORR and OER. Particularly, the NPC/FeCo@NCNT only needs an overpotential of 339.5 mV to deliver the current density of 10 mA cm^−2^ for OER and exhibits an onset potential of 0.92 V to drive ORR. More impressively, the NPC/FeCo@NCNT exhibits a bifunctional activity parameter (Δ*E*) as low as 0.741 V, indicating its bifuncational catalytic activity outperforms most of the state‐of‐the‐art bifunctional electrocatalysts reported. The experimental results and the density functional theory (DFT) calculations have demonstrated that the interplay between the FeCo NPs and the NCNT and the presence of N,P codoped carbon structure play important roles in the high catalytic performance of the NPC/FeCo@NCNT. Importantly, the NPC/FeCo@NCNT is demonstrated to have great potentials in improving the performance of liquid and all‐solid‐state zinc–air batteries.

## 2 Results and Discussion

### 2.1 Morphology Characterization and Formation Mechanism of the Catalysts

**Figure 1:**
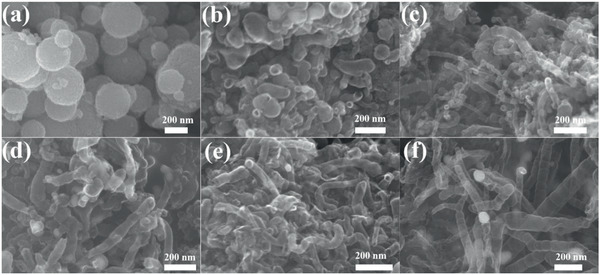
SEM images of the melamine–GO–PS–Fe–Co composite at different calcination stages: a) (420 °C, 2 h), b) (750 °C, 0 h), c) (750 °C, 0.1 h), d) (750 °C, 0.2 h), e) (750 °C, 0.3 h), and f) (750 °C, 0.5 h).

**Figure 2:**
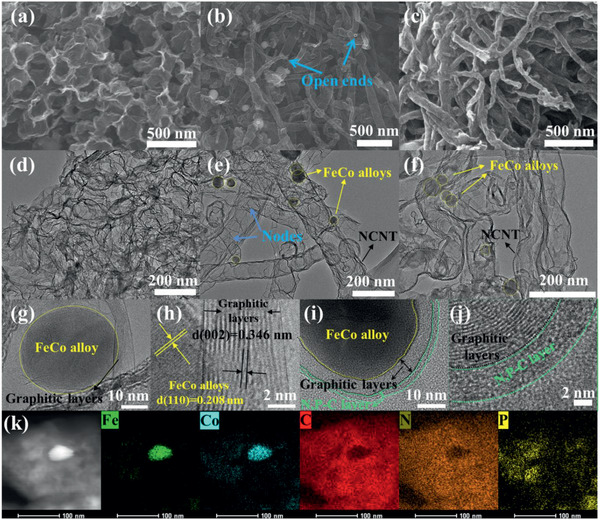
SEM images of a) NHGS, b) FeCo@NCNT, and c) NPC/FeCo@NCNT. d) TEM images of NHGS, e,g,h) FeCo@NCNT, and f,i,j) NPC/FeCo@NCNT. k) EDS elemental mapping images of NPC/FeCo@NCNT.

The scanning electron microscopy (SEM) image in **Figure** **1**a indicates that the melamine–GO–PS–Fe–Co composite used as the starting materials for the preparation of the NPC/FeCo@NCNT has a microspheric morphology. It consists of GO‐coated PS spheres with Fe^3+^, Co^2+^, and melamine adsorbed. The calcinations of the melamine–GO–PS–Fe–Co composite at the relatively low temperature of 420 °C produces the ball‐shaped materials with no fiber‐like products observed, as shown in Figure 1a. The evolution of the ball‐shaped materials gradually to the fiber‐like products can be clearly observed when it is calcined at the high temperature of 750 °C for 0, 0.1, 0.2, and 0.3 h, respectively, as shown by the SEM images in Figure 1b–e. The complete transformation of the ball‐shaped materials to the fiber‐like products is achieved when it is calcined at the high temperature of 750 °C up to 0.5 h. The Transmission electron microscopy (TEM) image in **Figure** **2**e indicates that these fiber‐like products are bamboo‐like tubularly structured with the embedment of the dark dots. The nodes of the bamboo‐like structure can be well observed in the TEM image, as indicated by the blue arrows in Figure 2e. The tubular structure of these fiber‐like products is also evidenced by their open ends as indicated by the arrow in Figure 2b. The transparent feature of these fiber‐like products, along with their tubular structure, makes us believe that they are nitrogen‐doped carbon nanotubes (NCNTs), which are well demonstrated by the structural characterizations shown below.

Figure 2h shows a high resolution TEM (HRTEM) image of the FeCo@NCNT, in which the lattice fringes corresponding to the (002) planes of the graphitic carbon can be observed. It also indicates that these dark dots are well crystallized with the lattice fringes clearly observable. 0.208 nm of the *d*‐spacing measured from the HRTEM image in Figure 2h is well consistent with the (110) planes of FeCo alloys. These results clearly demonstrate the formation of the FeCo@NCNT.

To understand the formation mechanism of the FeCo@NCNT, the same synthetic procedure for the synthesis of the FeCo@NCNT was performed without the addition of FeCl_3_ and Co(NO_3_)_2_ while keeping the other parameters unchanged. The result shows the formation of nitrogen doped hollow graphene spheres (NHGSs) with no fiber‐like products detected (Figure 2a). The production of these NHGSs can be a result of the decomposition of the PSs, the thermal reduction of the GO and the simultaneous doping of N from melamine into the graphitic structure of the reduced GO. The appearance of a large fraction of open hollow balls in Figure 2a indicates the break of the hollow structure by the gas stream generated from the decomposed PSs. These observations strongly support the important role of Fe^3+^ and Co^2+^ in the formation of the FeCo@NCNT. Previous work has proposed that the zero valent Fe and Co can act as the catalysts for the preparations of the CNTs.^[^ ^15^, ^16^ ^]^ In our work, Fe^3+^ and Co^2+^ can be reduced to the zero valent states during the high calcination temperature of 750 °C, which can act as the catalysts facilitating the transformation of the carbon sources to the CNTs. To further demonstrate the important role of Fe^3+^ and Co^2+^ in the formation of the FeCo@NCNT, the same synthetic procedure was also performed at the low and high concentrations of Fe^3+^ and Co^2+^. The results indicate that at the low concentrations of Fe^3+^ and Co^2+^, the obtained FeCo@NCNTs shows a similar structure, but with relatively shorter NCNTs (Figures S2a and S4, Supporting Information), while the high concentrations of Fe^3+^ and Co^2+^ produce the FeCo@NCNTs with relatively longer NCNTs (Figures S2b, S3, and S4, Supporting Information). This further validates the catalytic formation of the NCNTs by the zero valent Fe and Co from Fe^3+^ and Co^2+^. Additionally, control experiments also show that the absence of the PSs would lead to formation of nitrogen‐doped porous graphene supported FeCo NPs (FeCo@NPG) (Figure S5, Supporting Information) with no well‐defined NCNTs observed. The PSs used in our work are positively charged, which provides an appropriate surface for the adsorption of the negatively charged GO (due to the presence of oxygeneous groups, such as —OH, —COOH, and etc.) through the electrostatic interactions. This can well avoid the face‐to‐face aggregation of the GO and help the GO to expose more surface area for the adsorption of Fe^3+^, Co^2+^, and melamine. At the high calcination temperature, the PS decomposition will generate the carbon bearing gases. The thus‐formed gas stream can break and open the hollow structure of the GOs. The reaction of these carbon bearing gases under the catalysis of the zero valent Fe and Co produces the carbon materials, which help the GOs curl up forming the CNTs and produce the nodes in the CNTs. And meanwhile, the pyrolysis of melamine incurs the doping of N into the graphitic structure of the CNTs, facilitating the formation of the bamboo structured NCNTs.

The formation of the NPC/FeCo@NCNT was achieved by the calcination of the FeCo@NCNT in the presence of polyphosphazenes (Figure S1, Supporting Information). Figure 2c shows that the NPC/FeCo@NCNT inherits the morphology of the parent FeCo@NCNT. The TEM image in Figure 2f shows that the NPC/FeCo@NCNT consists of the tubularly structured materials with the embedment of the dark dots, which is very similar to the FeCo@NCNT. The HRTEM image in Figure 2j shows that in the case of the NPC/FeCo@NCNT a thin less‐crystallized NPC layer coated on the surface of the NCNTs could be observed. And meanwhile, the formation of the thin less‐crystallized NPC layer on the surface of the FeCo NPs can also be identified. These observations well demonstrated the successful preparation of the NPC/FeCo@NCNT.

### 2.2 Structure and Composition of the Catalysts

**Figure 3:**
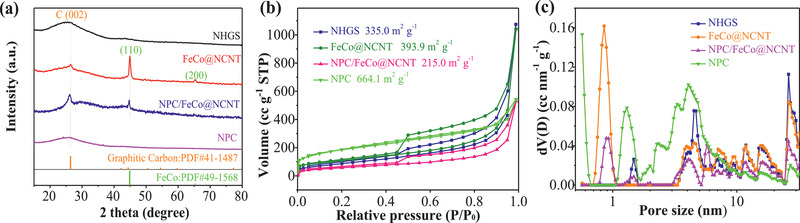
Structural characterizations of NHGS, FeCo@NCNT, NPC/FeCo@NCNT, and NPC. a) XRD patterns, b) N_2_ adsorption/desorption isotherm curves, and c) pore size distributions.

**Figure** **3**a displays the X‐ray powder diffraction (XRD) pattern of the FeCo@NCNT, which shows the diffraction peaks at 44.9° and 63.5°, ascribable to the (110) and (200) lattice planes of cubic bimetal FeCo alloy (JCPDS No. 49‐1568). A small peak at 26.5° can be ascribed to the diffractions from the (002) plane of well‐ordered graphitic carbon (JCPDS No. 41‐1487). These observations well demonstrates the formation of the FeCo@NCNT. The XRD pattern of the NPC/FeCo@NCNT exhibits the diffraction peaks similar to the FeCo@NCNT, but with the intensities of the peaks at 26.5°, 44.9°, and 63.5° relatively lower with respect to those of the FeCo@NCNT. This indicates the NPC coating does not alter the crystal phases of the FeCo@NCNT, but gives a blocking layer for the detection of the crystallinity of the FeCo@NCNT.

The N_2_ adsorption/desorption isotherms (Figure 3b) and the pore size distribution analysis (Figure 3c) indicate that the NPC/FeCo@NCNT has a porous structure with a high specific Brunauer‐Emmett‐Teller (BET) surface area. As shown in Figure 3b, the NPC/FeCo@NCNT exhibits type IV isotherms with obvious hysteresis loops in the P/P_0_ range of ≈0.4–1.0, indicating the coexistence of mesopores and macropores. The BET surface areas calculated based on the N_2_ adsorption/desorption isotherms in Figure 3b are 215.0 m^2^ g^−1^, which is slightly lower than that of the FeCo@NCNT. The decrease of the BET surface area of the NPC/FeCo@NCNT in comparison to that of FeCo@NCNT further supports the NPC coating. The hierarchical structure which coexists of microporous and mesoporous can give the NPC/FeCo@NCNT with sufficient surface area exposing more active sites^[^ ^9^, ^14^, ^17^ ^]^ and facilitate quick transport of relevant reactants at the triple‐phase interface during the catalytic processes,^[^ ^15^, ^18^ ^]^ which is helpful to improve the catalytic activities of the NPC/FeCo@NCNT.

**Figure 4:**
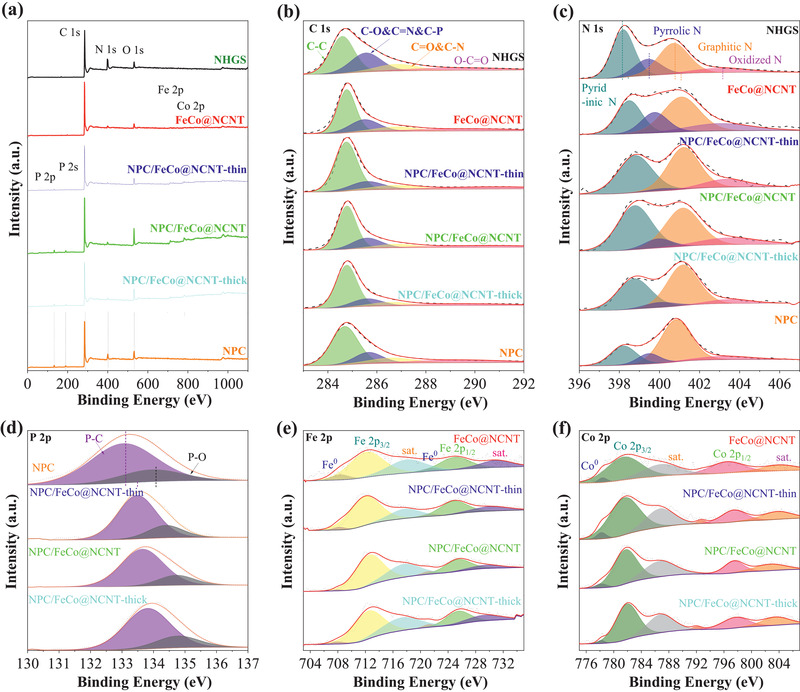
XPS spectra of NHGS, FeCo@NCNT, NPC/FeCo@NCNT‐thin, NPC/FeCo@NCNT, NPC/FeCo@NCNT‐thick, and NPC. a) Survey spectrum, b) C 1s, c) N 1s, d) P 2p, e) Fe 2p, and f) Co 2p.

To elucidate the surface chemical compositions and the electronic structures of the samples, the X‐ray photoelectron spectra (XPS) characterizations were performed. **Figure** **4**a and Table S1 (Supporting Information) indicate the presence of C, N, Fe, Co, and O in the FeCo@NCNT, while the presence of C, N, P, Fe, Co, and O in the NPC/FeCo@NCNT. In the both cases, the total contents of Fe and Co estimated by the XPS are ≈1.84 wt%. However, the weight percentages of the FeCo alloy in the NPC/FeCo@NCNT based on thermogravimetric analysis (TG) is 6.4 wt% as shown in Figure S6 of the Supporting Information. The metal content from XPS is far below the weight percentages of the FeCo alloy measured by TG analysis. This difference well demonstrates that the FeCo alloy NPs in the NPC/FeCo@NCNT are embedded in the NCNT. Since the embedment makes the FeCo alloy NPs less detectable by the XPS, due to the limited penetration capability of the X‐ray.^[^ ^19^ ^]^ Additionally, the relatively low weight percentage of the FeCo alloy NPs in the NPC/FeCo@NCNT, in comparison to that of the FeCo alloy NPs in the FeCo@NCNT, further demonstrates that the coating of the additional carbon layer on the surface of FeCo@NCNT.

Figure 4e exhibits the Fe 2p spectrum of the NPC/FeCo@NCNT. It indicates the presence of the two peaks centered at ≈725.6 and ≈712.7 eV, assignable to Fe 2p 1/2 and Fe 2p 3/2 of Fe^2+^/Fe^3+^, respectively, and the two peaks at ≈717.9 and ≈730.0 eV, assignable to the satellite peaks of Fe 2p 3/2 and Fe 2p 1/2, respectively. The peaks at ≈707.9 and ≈721.5 eV well demonstrate the presence of the zero valent Fe in the NPC/FeCo@NCNT. Analogously, the Co 2p spectrum (Figure 4f) also displays two obvious peaks at ≈797.5 eV (Co 2p 1/2) and ≈781.8 eV (Co 2p 3/2), assignable to Co 2p 1/2 and Co 2p 3/2 of Co^2+^/Co^3+^, respectively, and the two peaks at ≈802.9 and ≈787.0 eV, assignable to the satellite peaks of Co 2p 1/2 and Co 2p 3/2, respectively. The peaks at ≈778.2 and ≈792.8 eV demonstrate the presence of the zero valent Co in the NPC/FeCo@NCNT. It is worthwhile that in both the Fe 2p and Co 2p spectra, the peaks corresponding to the zero valent metals are much smaller than those at the ionic state of Fe^2+^/Fe^3+^ and Co^2+^/Co^3+^. This could be attributed to the high susceptibility of the small FeCo NPs to air and surface oxidization, making the surface metallic atoms mainly exist in the form of the high valent states, and the low permeability of the X‐ray, making the zero valent Fe and Co in the bulk of the FeCo NPs less detectable by XPS. This is consistent with the FeCo‐encapsulated carbon materials reported previously.^[^ ^16^, ^20^ ^]^

The O 1s spectrum of the NPC/FeCo@NCNT is given in Figure S7 of the Supporting Information. The curve fitting shows the domination of the peaks at 530.0, 531.3, 532.6, and 533.3 eV, corresponding to the Fe/Co—O (O_Lattice_), surface adsorbed O_2_, H_2_O(adsorbed)/C=O, and O—C—O/N—O, respectively. The extremely low intensity of the peak corresponding to oxygen bonded to metal (Fe/Co—O) well demonstrates that the low contents of Fe/Co—O bonds at the surface of the FeCo NPs and the FeCo NPs are embedded in the carbon materials (Table S2, Supporting Information). Worth noting is that although the curve fitting of the O 1s spectrum of the FeCo@NCNT exhibit the peaks at 530.0, 531.3, 532.6, and 533.3 eV, its intensity of the peak corresponding to the surface adsorbed O_2_ is much lower than that of the NPC/FeCo@NCNT and the intensities of the peaks corresponding to O—C—O/N—O and Fe/Co—O are much higher than that of the NPC/FeCo@NCNT. This further demonstrates the NPC coating on the surface of the NPC/FeCo@NCNT. The relatively higher intensity of the peak corresponding to the surface adsorbed O_2_ suggests a high oxyphilic nature of the NPC/FeCo@NCNT. This might be a fascinating feature of the NPC/FeCo@NCNT for its application as an electrocatalyst toward ORR, because the adsorption of oxygen on the surface of electrocatalyst has been recognized as an important step during the ORR. Most interestingly, the O 1s spectrum of the NHGS also exhibit peaks corresponding to the surface adsorbed O_2_, H_2_O (adsorbed)/C=O, and O—C—O/N—O, respectively. However, these peaks appear at positions with binding energies slightly lower than those of the NPC/FeCo@NCNT and the FeCo@NCNT. This strongly suggests that there exists a strong electronic coupling between the FeCo NPs and the carbon materials in both the NPC/FeCo@NCNT and the FeCo@NCNT with electron transfer from the FeCo NPs to the carbon materials. This observation is well consistent with the DFT calculations shown below.

The high‐resolution C 1s spectrum (Figure 4b) reveals the presence of C—C (≈284.8 eV), C‐O&C=N&C‐P (≈285.6 eV), C=O&C‐N (≈286.8 eV) and O—C=O (≈290 eV) (Table S3, Supporting Information), indicating that the N or/and P atoms are successfully incorporated into carbon skeleton. Figure 4c shows that the high‐resolution XPS N 1s spectrum of the NPC/FeCo@NCNT. Based on different binding energy values, it can be divided into four dominant characteristic peaks, indexable to the pyridinic N (398.77 eV), pyrrolic N (399.94 eV), graphitic N (401.16 eV), and oxidized N (403.51 eV), respectively. Obviously, the pyridinic N and graphitic N are major components, which account for ≈80% of nitrogen in the NPC/FeCo@NCNT (Table S4, Supporting Information). Previous work has proposed that the pyridinic N is an active site for ORR/OER, because it can increase the surface wettability of electrocatalysts,^[^ ^21^ ^]^ leading to more effective contact with reactants and better combination with adjacent C and FeCo atoms (acting as “bridge”) due to its great electron‐donating ability and metal‐coordination capability. This can optimize the surrounding chemical/electronic environment to promote the ORR/OER kinetics.^[^ ^22^ ^]^ The graphitic‐N is conductive to accelerate electron transfer and improve the limiting current densities of the ORR/OER processes.^[^ ^21^, ^23^ ^]^ So, we believe that such a nitrogen oxidation environment is beneficial to improve the catalytic activities of the NPC/FeCo@NCNT for the ORR/OER. Additionally, based on the N 1s spectrum shown in Figure 4c, a strong electronic coupling between the FeCo NPs and the carbon material can be inferred. As shown in Figure 4c, all the peaks of the N components in the FeCo@NCNT shifts to higher binding energies in comparison to those of the N components in the NPC (synthesized by the direct calcination of polyphosphazenes, Table S4, Supporting Information), implying an electron transfer from the FeCo NPs to the carbon material.

Figure 4d shows the P 2p spectrum of the NPC/FeCo@NCNT. The spectra fitting shows two components at the binding energies of ≈133.6 eV (P—C) and ≈134.7 eV (P—O). The relative ratio of the peak areas for P‐C and P‐O is 4:1 (Table S5, Supporting Information), which indicates that the P atom has been successfully doped into the carbon framework and exists mainly in the form of P—C. Previous reports indicates that the abundant P—C can be deemed as catalytically active sites to accelerate the kinetics of the ORR/OER.^[^ ^8^, ^24^, ^25^ ^]^ This suggests that the NPC/FeCo@NCNT can be used as the potential catalyst for the ORR/OER. Similar to the N 1s spectrum, careful analysis shows that all the P components in the NPC/FeCo@NCNT show the peak shifts to higher binding energy compared to the NPC, further supporting the strong electronic coupling between the FeCo NPs and the carbon material.

### 2.3 Electrocatalytic Performance for OER

**Figure 5:**
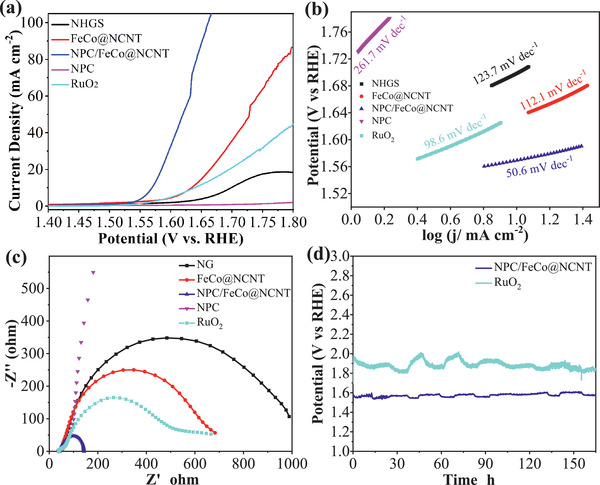
OER performance of NHGS, FeCo@NCNT, NPC/FeCo@NCNT, NPC, and RuO_2_ in 0.1 m KOH. a) OER polarization curves, b) Tafel plots, c) EIS Nyquist plots. d) Stability evaluation of NPC/FeCo@NCNT and RuO_2_ at 10 mA cm^−2^.

The OER performance of the samples was evaluated by the linear sweep voltammetry curves (LSVs) at a scan rate of 5 mV s^−1^ in 0.1 m KOH. **Figure** **5**a shows that the overpotential of the OER by the NPC/FeCo@NCNT at 10 mA cm^−2^ is 339.5 mV, which is smaller than those by NHGS (469.5 mV), FeCo@NCNT (404.5 mV), and NPC (>770 mV). This indicates that the NPC/FeCo@NCNT has the highest catalytic activity for OER among the catalysts reported in this work. Specifically, the catalytic activities of these catalysts follow the order of NPC/FeCo@NCNT >> FeCo@NCNT > RuO_2_ > NHGS > NPC. This observation well evidences the great importance of the FeCo alloy NPs and the NPC coating in the high OER catalytic activity of the NPC/FeCo@NCNT. It can be attributed to the reason that the strong coupling between interwoven NCNT network and incorporated binary FeCo alloy eases the electron transfer during the OER and endows the NPC/FeCo@NCNT with more active sites. Meanwhile, the NPC/FeCo@NCNT reported in this work has a fiber‐like structure, which facilitates the formation of a solid with the intertwined porous structure, shown in Figure 2c. This fiber‐like structure offers efficient pathways for adsorption and desorption relevant species such as O_2_, *OOH, *O, *OH, etc., which are favorable for enhancement of OER.^[^ ^3^, ^4^, ^26^ ^]^ The catalytic activity of the NPC/FeCo@NCNT even surpasses the commercial RuO_2_ (408.5 mV) (Figure 5a) and is also comparable to those of other state‐of‐art OER catalysts reported recently, such as, FeC_x_‐NC/CNTs‐10 (360 mV),^[^ ^27^ ^]^ FeCo/FeCoNi@NCNTs‐HF (378 mV),^[^ ^21^ ^]^ FeNi‐NC (380 mV),^[^ ^28^ ^]^ CoDNi‐N/C (360 mV),^[^ ^29^ ^]^ FeN*_x_*/PNC (395 mV),^[^ ^30^ ^]^ CoFe/N‐GCT (500 mV),^[^ ^31^ ^]^ etc. (Table S6, Supporting Information).

To further demonstrate the contribution of the NPC layer on the higher catalytic activity of the NPC/FeCo@NCNT, the catalytic activities of the NPC/FeCo@NCNT with a thin layer (NPC/FeCo@NCNT‐thin, Figure S8a, Supporting Information) and NPC/FeCo@NCNT with a thicker NPC layer (NPC/FeCo@NCNT‐thick, Figure S8b, Supporting Information) synthesized from the different ratios of polyphosphazenes and FeCo@NCNT are investigated. Figure S9 of the Supporting Information shows that the catalytic activities of the NPC/FeCo@NCNT‐thin and NPC/FeCo@NCNT‐thick also exceed that of FeCo@NCNT. This clearly demonstrates that the introduction of NPC can promote the OER catalytic activity. The thickness of the NPC layer can be a possible factor influencing the catalytic activity of the NPC/FeCo@NCNT. The thin NPC layer cannot completely cover the FeCo@NCNT, which leads to weak interaction between NPC and FeCo@NCNT, while the thicker NPC layer could completely block the NCNT component, which are unconducive to the exposure of N–C, FeCo, P–C active sites.^[^ ^23^ ^]^ As a consequence, the catalytic activity of the NPC/FeCo@NCNT follows the order: NPC/FeCo@NCNT > NPC/FeCo@NCNT‐thick > NPC/FeCo@NCNT‐thin > FeCo@NCNT. The strong coupling between FeCo@NCNT and NPC is also demonstrated to be indispensable to achieve high OER catalytic activities. This can be evidenced by the observation that the pure NPC (Figure S10, Supporting Information) exhibits extremely lower activity for the OER.

The Tafel plots were employed to evaluate the OER catalytic kinetics. Figure 5b shows that the NPC/FeCo@NCNT possesses much lower Tafel slope (50.6 mV dec^−1^) than those of NHGS (123.7 mV dec^−1^), FeCo@NCNT (112.1 mV dec^−1^), NPC (261.7 mV dec^−1^), and RuO_2_ (98.6 mV dec^−1^), suggesting a faster reaction kinetics of the OER by the NPC/FeCo@NCNT. This is well consistent with the results shown by the electrochemical impedance (EIS) spectra, which indicate that the NPC/FeCo@NCNT has lower charge transfer resistance than that of NHGS, FeCo@NCNT, NPC, and RuO_2_ (as demonstrated by its smaller diameter of the semicircles in the Nyquist diagram in Figure 5c. Additionally, the NPC/FeCo@NCNT is demonstrated to have a higher electrochemical active surface area (Figure S11, Supporting Information), as inferred from its higher double‐layer capacitance. Specifically, the double‐layer capacitance of NPC/FeCo@NCNT was calculated to be 20.87 mF cm^−2^, which is higher than that of the NHGS (6.41 mF cm^−2^), FeCo@NCNT (11.8 mF cm^−2^), and NPC (0.17 mF cm^−2^).

The durability test was performed to investigate the possibility of using the NPC/FeCo@NCNT for practical applications. Gratifyingly, the chronopotentiometric curve in Figure 5d shows that the NPC/FeCo@NCNT exhibits negligible potential loss even after 165 h of the OER, which strongly suggests its superb OER stability. Remarkably, the stability of the NPC/FeCo@NCNT surpasses than that of commercial RuO_2_ under the same conditions. The postelectrocatalysis analysis shows that the NPC/FeCo@NCNT after 165 h of the OER exhibits the XRD pattern identical to that of the original NPC/FeCo@NCNT (Figure S12, Supporting Information), which suggests that the crystal phases of the NPC/FeCo@NCNT are maintained during the OER. The SEM and TEM images in Figure S13 of the Supporting Information indicate the morphological preservation of the NPC/FeCo@NCNT after 165 h of the OER. The element mapping images well demonstrate that the FeCo alloy NPs are still covered by the NPC layer. These results strongly suggest that the specific structure, in which the FeCo NPs are embedded in the carbon materials, can well suppress the structural variation of the NPC/FeCo@NCNT during the complicated OER process, making the NPC/FeCo@NCNT potentially usable for practical applications.

### 2.4 Electrocatalytic Performance for ORR

**Figure 6:**
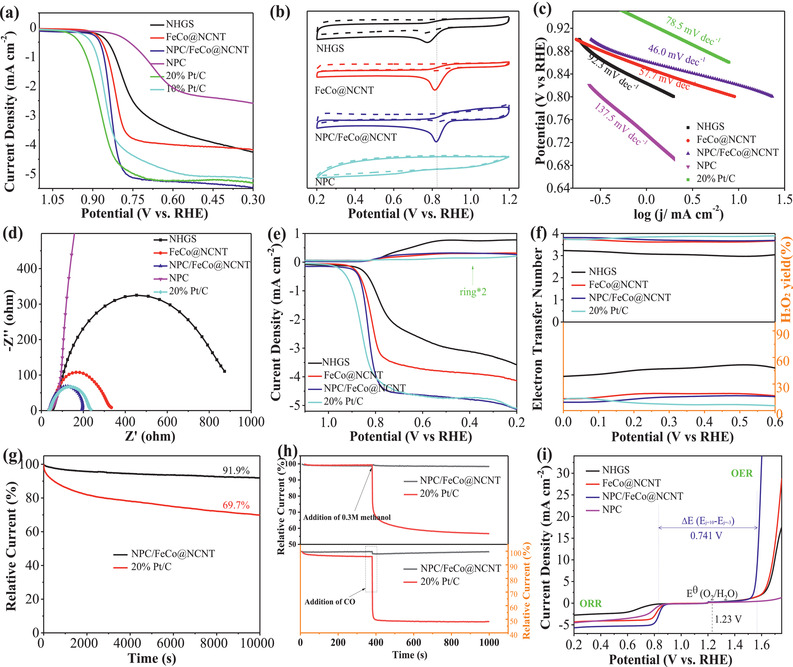
ORR performances of NGHSs, FeCo@NCNT, NPC/FeCo@NCNT, NPC, 10% Pt/C, and 20% Pt/C in O_2_‐saturated 0.1 m KOH. a) ORR polarization curves, b) Tafel plots, c) EIS Nyquist plots, d) CV curves (solid lines: in O_2_‐saturated 0.1 m KOH, dashed lines: in N_2_‐saturated 0.1 m KOH), e) RRDE polarization curves. f) Electron transfer numbers and yields of peroxide obtained from RRDE. g) Stability evaluation. h) Methanol and CO durability evaluation (the arrows indicate the addition of methanol and CO). i) Overall polarization curves of the ORR and OER for NHGS, FeCo@NCNT, NPC/FeCo@NCNT, and NPC.

The ORR catalytic activity of the NPC/FeCo@NCNT was evaluated in the O_2_‐saturated 0.1 m KOH using a rotating disk electrode. **Figure** **6**a shows that the ORR by the NPC/FeCo@NCNT exhibits an onset potential of 0.92 V and a limiting current density of ≈5.47 mA cm^−2^ (the LSVs of the ORR corrected by the iR drop given in Figure S14 of the Supporting Information shows a comparable result). Excitingly, both the ORR onset potential and limiting current density of the NPC/FeCo@NCNT are higher than those of NHGS (0.89 V, 4.05 mA cm^−2^), FeCo@NCNT (0.90 V, 4.16 mA cm^−2^), and NPC (0.83 V, 2.51 mA cm^−2^). Additionally, although the onset potential of NPC/FeCo@NCNT is still slightly inferior to 10 wt% Pt/C (0.95 V) and 20 wt% Pt/C (0.98 V), its limiting current density exceeds the commercial 10 wt% Pt/C (5.11 mA cm^−2^) and 20 wt% Pt/C (5.26 mA cm^−2^). These results well demonstrate that the NPC/FeCo@NCNT is a superior catalyst for the ORR. The catalytic activity of the NPC/FeCo@NCNT toward the ORR can be further demonstrated by comparison of its cyclic voltammograms (CVs) in the N_2_ and O_2_‐saturated 0.1 m KOH. As shown in Figure 6b, the CV of the NPC/FeCo@NCNT in the N_2_‐saturated solution exhibits as a featureless voltammetric curve with no peaks corresponding to the Faradaic reactions observed. This is unlike its CV in the O_2_‐saturated solution, where a distinct peak corresponding to the ORR can be identified. This result well demonstrates that the NPC/FeCo@NCNT is active for the ORR. Similar to that observed by the LSVs, the peak of the ORR by the NPC/FeCo@NCNT appears at the potential that is positive to those NHGS, FeCo@NCNT, and NPC, further evidencing that the NPC/FeCo@NCNT has high catalytic activity for the ORR than those of NHGS, FeCo@NCNT, and NPC.

The Tafel plots are used to evaluate the ORR kinetics. Figure 6c shows that the NPC/FeCo@NCNT exhibits a ORR Tafel slope of 46.0 mV dec^−1^. This value is lower than those of NHGS (92.3 mV dec^−1^), FeCo@NCNT (57.7 mV dec^−1^), NPC (137.5 mV dec^−1^), and 20 wt% Pt/C catalyst (78.5 mV dec^−1^), suggesting that the ORR by the NPC/FeCo@NCNT goes with a faster reaction kinetics. This result is well consistent with that obtained from the EIS spectra in Figure 6d. It shows that the NPC/FeCo@NCNT exhibits a smaller charge transfer resistance than that of NHGS, FeCo@NCNT, NPC, and Pt/C, suggesting a faster charge transfer rate during the ORR.

To clarify the influence of the NPC layer on the ORR catalytic activity of the NPC/FeCo@NCNT, we compared the catalytic activity of the NPC/FeCo@NCNT with those of the FeCo@NCNT, the NPC/FeCo@NCNT‐thin, and NPC/FeCo@NCNT‐thick. Figure S9b of the Supporting Information indicate that all the NPC/FeCo@NCNT with different NPC thicknesses exhibit higher catalytic activities for the ORR than that of the FeCo@NCNT. This result suggests that the NPC layer is indispensable in the high catalytic activity of the NPC/FeCo@NCNT. Additionally, similar to the case of the OER, the thickness of NPC layer is found to have a prefound influence on the ORR activities of the NPC/FeCo@NCNT. As shown in Figure S9b of the Supporting Information, the catalytic activity of the NPC/FeCo@NCNT is higher than those of both the NPC/FeCo@NCNT‐thin and the NPC/FeCo@NCNT‐thick. This observation well necessitates a moderate NPC thickness on the NPC/FeCo@NCNT to give a higher catalytic activity for the ORR.

The rotating ring‐disk electrode (RRDE) measurements were carried out to evaluate the electron transfer number (n) and the yield of hydrogen peroxide production (%H_2_O_2_) during the ORR and infer the ORR catalytic pathways (Figure 6e,f). The results show that the ORR by the NPC/FeCo@NCNT has the electron transfer numbers ranging from 3.67 to 3.82 and low yields of hydrogen peroxide production ranging from ≈9.0% to 16.5%, suggesting that the ORR by the NPC/FeCo@NCNT mainly proceeds via a four‐electron transfer pathway. Furthermore, the electron transfer numbers of the ORR by the NPC/FeCo@NCNT are higher than those of NHGS, FeCo@NCNT, and NPC, while its yields of hydrogen peroxide production are lower than those of NHGS, FeCo@NCNT, and NPC. This further evidences the higher catalytic activity of the NPC/FeCo@NCNT than those of NHGS, FeCo@NCNT, and NPC.

The LSVs of the ORR by the NPC/FeCo@NCNT were investigated by the rotating disk electrode at the different rotation speeds (400–2025 rpm) to gain further insight into the catalytic process. Figures S15 and S16 of the Supporting Information show the increase of the limiting current densities with increase of the rotation speed. Analysis of the LSVs at the different rotation speeds using the Koutecky–Levich plot shows that the ORR by the NPC/FeCo@NCNT has the kinetic current density (*j*_k_) of 25 mA cm^−2^, which far exceeds than that of NHGS, FeCo@NCNT, and NPC (Figure S15i, Supporting Information). This result is well in line with those obtained by the Tafel plots and EIS spectra, which shows that the ORR by the NPC/FeCo@NCNT goes with a faster reaction kinetic.

The durability test shows that the NPC/FeCo@NCNT can maintain its high catalytic activity for the ORR with <8.1% of the loss in its original activity after 10 000 s of the continuous working (Figure 6g). The stability of the NPC/FeCo@NCNT for the ORR is much higher than that of 20 wt% Pt/C, in which over 31% of the loss in its original activity is observed after 10 000 s of continuous working. Post‐electrocatalytic analysis by the SEM, TEM, elemental mapping, and XRD (Figures S17 and S18, Supporting Information) indicates that structure and morphology of the NPC/FeCo@NCNT can be well preserved during the ORR. This is beneficial from its specific structure, in which the FeCo NPs are embedded in the carbon material. So, the structure and morphology variations during the ORR can be well suppressed. Particularly, the NPC/FeCo@NCNT is also demonstrated to have good tolerance toward CO and methanol. As shown in Figure 6h, the introduction of CO and methanol has negligible effects on the catalytic activity of the NPC/FeCo@NCNT. This is unlike the Pt/C, by which an obvious of drop in its catalytic activity can be observed after the introduction of CO and methanol. The presence of the NPC layer is demonstrated to be helpful in improving the stability of the NPC/FeCo@NCNT. As shown in Figure S19 of the Supporting Information, the activity loss of the NPC/FeCo@NCNT (<8.1%) is slightly lower than that of the FeCo@NCNT(≈9.8%) after 10 000s of continuous working, although both the NPC/FeCo@NCNT and the FeCo@NCNT exhibit identical tolerance to CO and methanol. The high stability and good tolerance toward CO and methanol, along with the high catalytic activity, suggest that the NPC/FeCo@NCNT is usable as the efficient ORR catalyst for practical applications.

### 2.5 DFT Calculation

The results shown above well verifies that the NPC/FeCo@NCNT can be used as a highly efficient and stable catalyst for OER and ORR. This can be further demonstrated by its bifunctional catalytic performance assessed by the potential difference of ∆*E* = *E*_OER,_ *_j_*_= 10_ – *E*_ORR,_ *_j_*_= −3_, (where *E*_OER,_ *_j_*_= 10_ and *E*_ORR,_ *_j_* _= −3_ are the potentials of OER and ORR at the current densities of 10 and −3 mA cm^−2^, respectively. Basically, the smaller ∆*E* indicates a better ORR/OER bifunctional catalytic activity. Figure 6i shows that the NPC/FeCo@NCNT exhibits a smallest ∆*E* of 0.741 V among the catalysts reported in this work. Outstandingly, this value is even smaller than those of the Pt/C + RuO_2_ and most bifunctional catalysts reported recently (Table S6, Supporting Information), clearly demonstrating the superiority of the NPC/FeCo@NCNT as the bifunctional catalysts for the ORR/OER. Based on the results and analysis shown above, we believe that the interplay between the FeCo NPs and the carbon materials and the presence of N and P codoped structure play an important role in the high catalytic activity of the NPC/FeCo@NCNT. So, to well understand it, the DFT calculations were performed.

**Figure 7:**
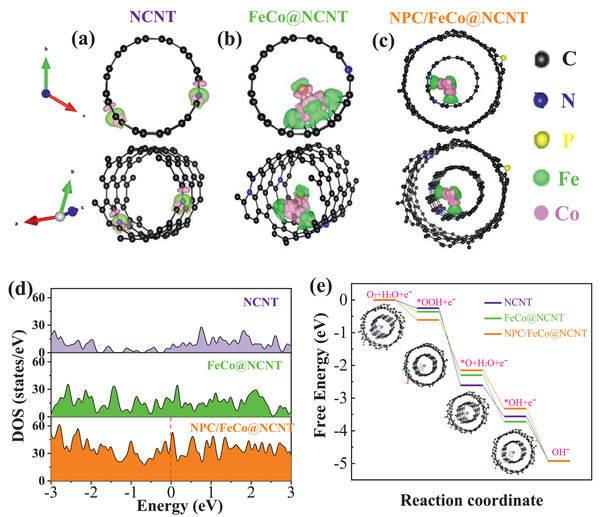
Theoretical studies of the crystal structures, electronic structure, free‐energy, and electron transfer properties of NCNT, FeCo@NCNT, and NPC/FeCo@NCNT. a–c) The optimized geometry models. d) The calculated density of states. e) Free‐energy change of the intermediates (*OOH, *O, and *OH) in the standard four electron transfer reaction pathway. The insets show the optimized adsorption geometry of O_2_, *OOH, *O, and *OH on the three electrocatalysts.

Three structures, including NCNTs, FeCo alloy NPs encapsulated in N‐doped carbon nanotubes (FeCo@NCNTs), and N,P codoped carbon layer coated on FeCo alloy NPs encapsulated NCNT (**Figure** **7**a–c), were established to model the ORR/OER on the NCNT, FeCo@NCNT, and NPC/FeCo@NCNT. Figure 7d shows that all the catalysts exhibit the ideal density of states crossing over the Fermi level, suggesting the high electric conductivity of these catalysts. Impressively, the electronic states around Fermi level are enhanced in the FeCo@NCNT compared with that of NCNT, indicating the improved metallic feature of the FeCo@NCNT. For the NPC/FeCo@NCNT, the metallic feature can be further increased due to the hybridization effect among 2p states of N and adjacent C atoms and 3d states of FeCo atoms, which gives it the sharper peaks near Fermi level. The charge distribution analysis shows that the coupling of the FeCo cluster with NCNT can enable direct electron transfer from FeCo to NCNT (Figure 7b,c). This is well consistent with the XPS results in Figure 4, which shows a strong electronic coupling between the FeCo nanoparticle and the carbon material with an electron transfer from the FeCo nanoparticle to the carbon material. This electron transfer, along with the N,P codoping, could increase delocalized “electron‐donating area,” facilitating the adsorption of the reactants and intermediates during the ORR/OER process and thereby improving the catalytic activities. As displayed in Figure 7e, for the ORR, all four elementary reaction pathways go downhill and represent exothermic reactions, which are regarded as follows: adsorbed O_2_ dissociation into *OOH (O_2_ (g) + H_2_O(l) + e^−^→*OOH + OH^−^); *OOH decomposition into *O (*OOH + e^−^→*O + OH^−^); *O alkalization into *OH (*O + H_2_O(l) + e^−^→*OH + OH^−^); *OH detachment into OH^−^ (*OH + e^−^→OH^−^). Among them, the process with the smallest Gibbs free energy change ΔG*_i_* (*i* = 1, 2, 3, 4) is the rate‐determining step. The smallest ΔG_1_ indicate that the first reaction step is the rate‐limiting step, where the ΔG_1_ values of rate limiting step are calculated to be 0.25 eV (NCNT), 0.36 eV (FeCo@NCNT), and 0.61 eV (NPC/FeCo@NCNT), suggesting that the ORR activities increase in the order of: NCNT < FeCo@NCNT < NPC/FeCo@NCNT. This is in good accordance with the experimental results. On the contrary, for the OER, it is the reverse reaction of ORR and all four reaction pathways are endergonic, therefore, the most uphill step is the rate‐determining step. The biggest Gibbs free energy change values of NCNT, FeCo@NCNT, and NPC/FeCo@NCNT are 2.36, 1.94, and 1.6 eV, respectively. Obviously, the NPC/FeCo@NCNT also exhibits the best catalytic activity for OER. These DFT calculation results clearly demonstrate that the high catalytic activities of the NPC/FeCo@NCNT for the ORR/OER are the result of the interplay between the FeCo NPs and the carbon materials and the presence of N and P codoped structure.

### 2.6 Studies of the Liquid and All‐Solid‐State Zinc–Air Batteries

**Figure 8:**
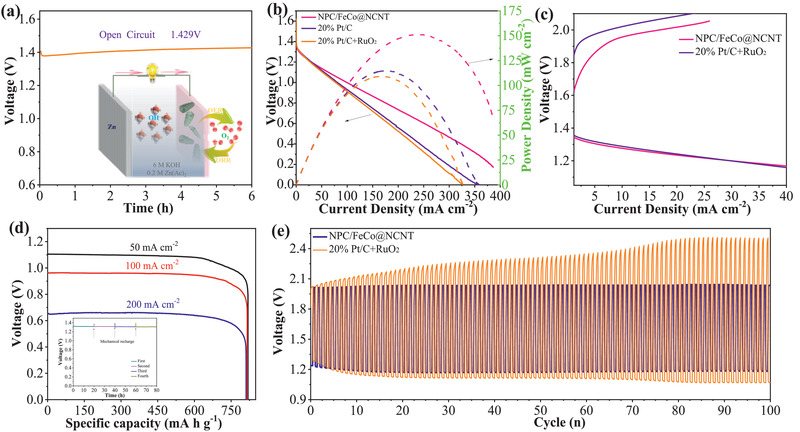
ZAB performance of NPC/FeCo@NCNT and 20% Pt/C + RuO_2_: a) OCP measurement of NPC/FeCo@NCNT assembled ZAB (Inset is the schematic illustration of the ZABs.). b) Discharge polarization and power density curves of ZABs with NPC/FeCo@NCNT, 20% Pt/C, and 20% Pt/C + RuO_2_ with WT_20% Pt/C_:WT${}_{{\text{RuO}}_{2}}$ = 1:1. c) Charge and discharge polarization curves. d) Specific discharging capacity plots at 50, 100, and 200 mA cm^−2^. Inset is the long‐time durability of ZABs based with NPC/FeCo@NCNT (the number represents the different mechanical recharge cycles). e) Galvanostatic charge–discharge cycling curves of the ZAB at 10 mA cm^−2^.

Encouraged by its excellent bifunctional catalytic activities of the NPC/FeCo@NCNT for ORR and OER, we evaluated the practical applications of the NPC/FeCo@NCNT as the air‐electrode catalyst in the home‐made rechargeable liquid ZABs. **Figure** **8**a shows that the open‐circuit potential (OCP) of the ZAB based on the NPC/FeCo@NCNT is 1.429 V, which is higher than that of the ZAB with the FeCo@NCNT (1.40 V, Figure S20, Supporting Information). Figure 8b indicates that the ZAB with the NPC/FeCo@NCNT can exhibit a maximum power density of 151.3 mW cm^−2^ at 239.6 mA cm^−2^, which is 30% higher than that of the ZAB with the FeCo@NCNT (115.9 mW cm^−2^, Figure S21, Supporting Information). Remarkably, the maximum power density of the ZAB with the NPC/FeCo@NCNT is also 27% and 38% higher than those of the ZABs with 20% Pt/C (118.3 mW cm^−2^) and 20% Pt/C + RuO_2_ (109.6 mW cm^−2^) at the same catalyst mass loadings, respectively. Additionally, the charge and discharge polarization curves show that the ZAB with the NPC/FeCo@NCNT exhibits a lower potential gap for charging and discharging than that of 20% Pt/C + RuO_2_ (Figure 8c), suggesting a better reversibility of the NPC/FeCo@NCNT than that of 20% Pt/C + RuO_2_. Table S7 of the Supporting Information indicates that the maximum power density of the ZAB with the NPC/FeCo@NCNT even exceeds those of the ZABs reported recently.

Figure 8d shows that the ZAB with the NPC/FeCo@NCNT can exhibit stable discharge platforms even at big current densities from 50 to 200 mA cm^−2^. By normalizing the weight of consumed Zn, its specific capacities are estimated ≈818, 815, and 810 mAh g_Zn_^−1^ at big current densities of 50, 100, and 200 mA cm^−2^, respectively, achieving ≈99% utilization of the theoretical capacity (820 mAh g_Zn_^−1^). Although Zn metal is consumed during the discharge process, the ZAB battery can be mechanically recovered by replacing the Zn plate and electrolyte. As expected, the ZABs based on NPC/FeCo@NCNT can continue to work without any potential decrease after more than 80 hours of operation (the inset of Figure 8d), demonstrating that the NPC/FeCo@NCNT is stable in the ZAB working environment and the dissolved zinc ion will not deposit on the catalyst and reduce the activity of the catalyst.^[^ ^24^ ^]^

The long‐life cycling stability and efficiency of the ZAB with the NPC/FeCo@NCNT was also assessed by galvanostatic discharge/charge cycles at 10 mA cm^−2^. Figure 8e shows that the initial charge and discharge potentials for the ZAB with NPC/FeCo@NCNT are 2.06 and 1.26 V, respectively, with the high round‐trip efficiency of 62.2%. Even after continuous working for 100 cycles, no obvious voltage drop is observed and the voltaic efficiency maintains 59.3%. This is contrast to the ZABs with the 20% Pt/C + RuO_2_, which exhibits a huge discharge potential reduction and charge potential increment under the same condition. Furthermore, a clear efficiency decrease from 61.3% to 42.8% could be observed in the ZABs with the 20% Pt/C + RuO_2_, presumably due to the agglomeration of Pt or RuO_2_ NPs and the carbon corrosions.^[^ ^32^ ^]^ These results further demonstrate that the NPC/FeCo@NCNT is highly stable in the ZAB working environment. Obviously, the excellent stability of the ZAB with the NPC/FeCo@NCNT is attributable to the high stability of the NPC/FeCo@NCNT, which can maintain its structural robustness during the OER and ORR processes, as demonstrated above.

**Figure 9:**
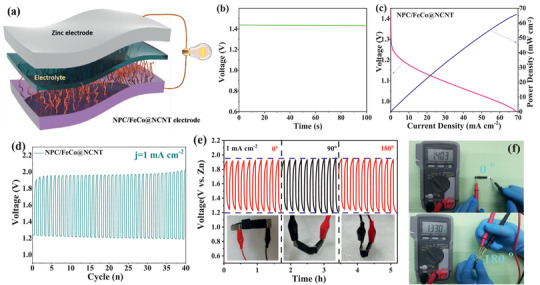
Application of NPC/FeCo@NCNT as air cathode in ASS‐ZABs. a) schematic illustration, b) open‐circuit voltage, c) discharge polarization curves and power density, d) galvanostatic discharge and charge cycling curves at 1 mA cm^−2^, e) discharge/charge curves of NPC/FeCo@NCNT‐based ASS‐ZAB under different bending states, and f) digital images with open‐circuit potential of the batteries at flat and folded states.

The NPC/FeCo@NCNT is further used to investigate its potential applications in the rechargeable flexible all‐solid‐state Zn–air batteries (ASS‐ZABs, **Figure** **9**a). Figure 9b shows that the ASS‐ZABs with the NPC/FeCo@NCNT can exhibit an open‐circuit potential as high as 1.45 V. The value is higher than those of most ASS‐ZABs reported previously (Table S7, Supporting Information). The discharge polarization curve in Figure 9c indicates that the ASS‐ZABs with the NPC/FeCo@NCNT can deliver the discharging potentials of 1.21, 1.15, and 1.10 V at 5, 15, and 30 mA cm^−2^, respectively. A high power density of 65.0 mW cm^−2^ can still be achieved when the ASS‐ZABs with the NPC/FeCo@NCNT is discharged at 70 mA cm^−2^. This value is higher than those of the ZABs with the state‐of‐art catalysts, such as NGM‐Co (28 mW cm^−2^),^[^ ^33^ ^]^ CoN_4_/NG (28 mW cm^−2^),^[^ ^34^ ^]^ (Zn,Co)/NSC (15 mW cm^−2^),^[^ ^35^ ^]^ and IOSHs‐NSC (60 mW cm^−2^),^[^ ^36^ ^]^ indicating that the NPC/FeCo@NCNT is a competitive catalyst for the practical applications in the ASS‐ZABs. Figure 9d shows no obvious voltage gap increase when charged/discharged up to 40 cycles at 1 mA cm^−2^, suggesting the good stability of the ASS‐ZABs with the NPC/FeCo@NCNT. Besides, the charge and discharge potentials of the as‐assembled battery exhibit a small gap and remain almost unchanged, even when the battery is tested under various bending conditions (Figure 9e). This is very comparable to the state‐of‐art air electrodes reported in the literature.^[^ ^12^, ^37^ ^]^ Furthermore, Figure 9f shows that the ASS‐ZABs can exhibit a voltage retention of 95% when bended from initial 0° (1.403 V) to 180° (1.330 V), suggesting its good flexibility stability. The more impressive demonstration of the NPC/FeCo@NCNT for practical applications is that two NPC/FeCo@NCNT assembled ZABs connected in series can electrically power the LEDs (Figure S22, Supporting Information) and rotate a hand shaped toy (Video S1, Supporting Information). These exciting results well verify that the NPC/FeCo@NCNT is potentially usable in the ASS‐ZABs for the applications in flexible and smart devices.

## 3 Conclusion

In summary, a novel approach has reported for the preparation of the NPC/FeCo@NCNT with a specific structure, in which the FeCo NPs is embedded in the NPC‐coated bamboo‐like NCNTs. Specifically, the preparation of the NPC/FeCo@NCNT is achieved by the calcination of the GO‐coated PS spheres with Fe^3+^, Co^2+^, and melamine adsorbed, during which the decomposition of the PSs generates the carbon bearing gases. The reaction of the carbon bearing gases under the catalysis of the zero valent Fe and Co produces the carbon materials, which help the GOs curl up forming the CNTs and produce the nodes in the CNTs. The pyrolysis of melamine induces the doping of N into the graphitic structure of the CNTs, leading to the formation of the bamboo‐structured NCNTs. When employed as the bifunctional catalyst, the NPC/FeCo@NCNT only needs an overpotential of 339.5 mV to deliver the current density of 10 mA cm^−2^ for OER and an onset potential of 0.92 V to drive ORR. Its bifuntional catalytic activities for OER and ORR are higher than those of the composite catalyst of Pt/C + RuO_2_ and surpass those of most bifuncational catalysts reported. The interplay between the FeCo NPs and the carbon materials and the presence of N and P codoped carbon structure are demonstrated to be the crucial factors improving the catalytic performance of the NPC/FeCo@NCNT. Additionally, the NPC/FeCo@NCNT shows the great potentials for practical applications to improve the performance of liquid and all‐solid‐state zinc–air batteries.

## Conflict of Interest

The authors declare no conflict of interest.

## Supporting information



Supporting Information

Supplemental Video 1

## Data Availability

Research data are not shared.
