# Supplementary material for: N‐Doped Carbon Nanotubes Derived from Graphene Oxide with Embedment of FeCo Nanoparticles as Bifunctional Air Electrode for Rechargeable Liquid and Flexible All‐Solid‐State Zinc–Air Batteries
Source: Adv Sci (Weinh). 2021 Mar 16;8(10):2004572. doi: 10.1002/advs.202004572 (PMC8132152; doi:10.1002/advs.202004572)
Supplement: Supplementary file 1 — Supporting Information [file ADVS-8-2004572-s001.pdf]

© 2021 Wiley-VCH GmbH

ADVANCED SCIENCE
Open Access

Supporting Information

for *Adv. Sci.,* DOI: 10.1002/advs.202004572

N-doped Carbon Nanotubes Derived from Graphene Oxide with Embedment of FeCo Nanoparticles as Bifunctional Air Electrode for Rechargeable Liquid and Flexible All- Solid-State Zinc-Air Batteries

Xiaoqiong Hao, Zhongqing Jiang, Baoan Zhang, Xiaoning Tian, Changsheng Song,* Likui Wang,* Thandavarayan Maiyalagan, Xiaogang Hao,* and Zhong-Jie Jiang**

Copyright WILEY-VCH Verlag GmbH & Co. KGaA, 69469 Weinheim, Germany, 2016.

Supporting Information

N-doped Carbon Nanotubes Derived from Graphene Oxide with Embedment of FeCo Nanoparticles as Bifunctional Air Electrode for Rechargeable Liquid and Flexible All-Solid-State Zinc-Air Batteries

Xiaoqiong Hao, Zhongqing Jiang, Baoan Zhang, Xiaoning Tian, Changsheng Song,* Likui Wang,* Thandavarayan Maiyalagan, Xiaogang Hao,* and Zhong-Jie Jiang**

Dr. X. Hao, Prof. Z. Jiang, B. Zhang, Dr. C. Song
Key Laboratory of Optical Field Manipulation of Zhejiang Province, Department of Physics, Zhejiang Sci-Tech University, Hangzhou 310018, P. R. China.
E-mail: zhongqingjiang@zstu.edu.cn; cssong@zstu.edu.cn.
Dr. X. Hao, Prof. X. Hao
Department of Chemical Engineering, Taiyuan University of Technology, Taiyuan 030024, P. R. China.
E-mail: xghao@tyut.edu.cn.
Dr. X. Tian
Department of Materials and Chemical Engineering, Ningbo University of Technology, Ningbo 315211, P. R. China.
Prof. L. Wang
The Key Laboratory of Synthetic and Biological Colloids, Ministry of Education, School of Chemical and Materials Engineering, Jiangnan University, Wuxi, P. R. China.
E-mail: lkwang@jiangnan.edu.cn
Dr. T. Maiyalagan
Electrochemical Energy Laboratory, Department of Chemistry, SRM Institute of Science and Technology, SRM Nagar, Kattankulathur, 603203, India.
Prof. Z.-J. Jiang
Guangdong Engineering and Technology Research Center for Surface Chemistry of Energy Materials & Guangzhou Key Laboratory for Surface Chemistry of Energy Materials, New Energy Research Institute, College of Environment and Energy, South China University of Technology, Guangzhou 510006, P. R. China.
E-mail: eszjiang@scut.edu.cn.

This PDF file includes:

Experimental Section
Theoretical calculation details
Figures. S1 to S22
Tables S1-S7

Experimental Section

1. Chemical Materials: Flake graphite (325 meshes) was bought from Alfa Ltd. Concentrated sulfuric acid (H_2SO_4, 96.0%), sodium nitrate ($NaNO_3$, ≥99.0%), hydrochloric acid (HCl, 37.0%), potassium hydroxide (KOH, ≥85.0%), potassium permanganate ($KMnO_4$, ≥99.5%), hydrogen peroxide aqueous solution (H_2O_2, 30.0%), 2,2'-Azobis (2-methylpropionamidine) dihydrochloride (97.0%), polyvinyl pyrrolidone (K-30), styrene (C_8H_8, ≥99.5%), melamine ($C_3H_6N_6$, ≥99.0%), cobalt nitrate hexahydrate ($Co(NO_3)_2{\cdot}6H_2O$), ≥98.5%), iron chloride anhydrous ($FeCl_3$, ≥97%), zinc acetate ($Zn(Ac)_2$, ≥99.0%), urea (H_2NCONH_2, ≥99.0%), hydrazine hydrate ($N_2H_4{\cdot}H_2O$, ≥85.0%), phosphonitrilic chloride trime ($Cl_6N_3P_3$, 98%), 1,4-dioxane (≥99.0%), triethylamine (≥99.0%), para-henylenediamine (97%), polyvinyl alcohol (PVA, 99.0%), isopropanol ($CH_3CH(OH)CH_3$, ≥99.5%) and methanol (CH_3OH, 99.5%) were bought from Shanghai Chemical Reagent Co. Ltd. Carbon paper (CP), Carbon cloth, commercial 20% Pt/C were purchased from Shanghai Hesen Electric Co., Ltd. (Shanghai, China). Zinc foil (99.994%) was purchased from Alfa Aesar. Nafion (5.0 wt.%) was purchased from DuPont Company. All the chemicals were used as received without further purification. Deionized (DI) water (H_2O) through Millipore system (Milli-Q®) was used in all the experiments.

2. Preparation of FeCo@NCNT, NPC/FeCo@NCNT and NHGS: Polystyrene spheres as the hard templates (PSs) were prepared as our previous work.[1] To make them positively charged, 2 g of the PSs were added to 100 mL 0.5 M HCl and stirred for 0.5 h. 50 mL of 2 mg mL^{-1} negatively charged GO solution (prepared using the methods reported elsewhere[1]) was then added and stirred for 12 h. This led to the wrapping of the GO layer onto the surface of PS. After that, 3 g melamine was added and stirred for another 12 h. Subsequently, 2 mL of 2 mM $FeCl_3$ and 2 mL of 2 mM $Co(NO_3)_2$ were added and continuously stirred for 96 h, followed by the addition of 0.1 mL hydrazine hydrate and 0.25 mol urea. The resulting

mixture was heated up to 110 °C and reacted for 24 h. After freeze-drying, the as-collected solid, along with the addition of 1.2 g melamine, was redissolved in 100 mL H_2O and stirred for 12 h. Afterward, the dried product was calcinated at 420 and 750°C for 2 h and 0.5 h in N_2 atmosphere, respectively. The thus-obtained FeCo@NCNT was successively washed by 100 mL of 2 M H_2SO_4 at 80 °C for 8 h and DI water for several times. For comparison, the nitrogen-doped hollow graphene spheres (NHGSs) was also synthesized without the addition of $FeCl_3$ and $Co(NO_3)_2$ but keeping the other experimental parameters constant. To verify the contribution of FeCo alloy on the formation of CNT, the FeCo@NCNTs were also synthesized by the addition of 1 mL 2 mM $FeCl_3$ and 1 mL 2 mM $Co(NO_3)_2 \cdot 6H_2O$, 3 mL 2 mM $FeCl_3$ and 3 mL 2 mM $Co(NO_3)_2 \cdot 6H_2O$, respectively, with the other experimental parameters constant. In addition, to verify the contribution of PSs on the formation of CNT, FeCo@NPG was also synthesized without the addition of the PSs as the hard templates.

For preparation of polyphosphazenes, 5.18 g phosphonitrilic chloride trime and 4.82 g para-phenylenediamine were dissolved successively in 100 mL of 1,4-dioxane with stirring to form solution A, and then 9.02 g triethylamine was dissolved in another 100 mL of 1,4-dioxane with stirring to form solution B. The solution A was mixed with the solution B under stirring, and then heated up to 70 °C for 12 h. The obtained product was collected by centrifugation and washed with 0.5 M H_2SO_4 and ethyl alcohol for several times, and then dried at 70 °C overnight for further use. The schematic illustration of the preparation of polyphosphazenes is shown in **Figure S1**. The obtained polyphosphazenes was pyrolysis at 800 °C for 2 h under N_2 atmosphere to obtain N, P co-doped carbon (NPC).

For the preparation of NPC/FeCo@NCNT, 40 mg FeCo@NCNT and 40 mg of polyphosphazenes were grinded, and then subjected to pyrolysis at 800 °C for 2 h with a heating rate of 5 °C min^{-1} under N_2 atmosphere. To further demonstrate the contribution of the NPC layer on the higher catalytic activity of the NPC/FeCo@NCNT, the samples of the NPC/FeCo@NCNT with a thin NPC layer (NPC/FeCo@NCNT-thin) and the

NPC/FeCo@NCNT with a thick NPC layer (NPC/FeCo@NCNT-thick) were synthesized from 20 mg and 80 mg of polyphosphazenes, respectively.

3. Characterization: The morphologies of obtained samples were characterized using scanning electron microscopy (SEM, Model Quanta 650 FEG) with an operation voltage of 20.0 kV. Transmission electron microscopy (TEM) and high resolution TEM (HRTEM) were performed on JEM-2100F at an accelerate voltage of 200 kV. Elemental mappings were obtained by energy-dispersive X-ray spectroscopy (EDS) with SEM. The structure analysis was performed by X-ray diffractometer (XRD) using Cu Kα radiation (10° - 80°, 5° min^{-1}). The Brunauer-Emmett-Teller (BET) specific surface area were detected by Quantachrome AutoSorb iQ_2 instrument at 77 K. The pore-size distribution was obtained from the QSDFT method. The composition and chemical states were determined by X-ray photoelectron spectroscopy (XPS) on Thermo VG Scientific ESCALAB 250 spectrometers with Al Kα radiation.

4. Electrochemical Measurements: All the OER and ORR tests were performed at room temperature using CHI 760E electrochemical workstation (Shanghai Chenhua Co., China) equipped with WaveVortex 10 Electrode Rotator (Pine Research Instrumentation, USA). The traditional three-electrode system was employed, including the polished glassy carbon rotating disk electrode (GC, 5 mm in diameter, 0.19625 cm^2) loaded with the obtained catalysts as working electrode, the saturated calomel electrode (SCE) and Pt wire serve as reference and counter electrode, respectively. The electrocatalyst inks were prepared by dispersing 4 mg as-prepared sample in 652 μL deionized water, 87 μL 5 wt% Nafion, 261 μL isopropanol mixture solution via continuous sonication for 1 h. 10 μL as-prepared ink was pipetted onto the polished GC electrode with a mass loading ~ 0.2 mg cm^{-2}, then dried at ~ 40 °C. All measured potentials were converted to the standard reversible hydrogen electrode

(RHE) calculated by: E(RHE) = E(SCE) + 0.059 pH + 0.2415. The current densities were normalized with the geometric surface area.

All the OER tests were carried out in 0.1 M KOH solution. The OER linear polarization curves (LSVs) were recorded at scan rate 5 mV s^{-1}. The Tafel slopes were calculated by LSV results using the Tafel equation as: $\eta = a + b \log(j)$, where η, a, b and j are the overpotential, Tafel constant, Tafel slope and measured current density, respectively. The overpotential (η) were obtained by the equation: η (V) = E (vs. RHE) - 1.23 V. The electrochemical impedance spectroscopy (EIS) measurements were performed with the frequency range of 0.01-100000 Hz at 1.53 V (vs. RHE). Furthermore, the OER stability was evaluated by potential-time chrono-potentiometric measurements at 10 mA cm^{-2} for 165 h. For comparison, the commercial RuO_2 catalyst electrodes were also tested.

For the ORR performances, all the measurements were carried out in 0.1 M KOH solution. The Tafel slopes calculation is the same as OER. The cyclic voltammetry (CV) curves were tested in N_2 and O_2 saturated 0.1 M KOH at a scan rate of 10 mV s^{-1}. The EIS were performed at 0.85 V (vs. RHE) with the frequency range of 0.01 -100000 Hz.

The LSVs were performed at 5 mV s^{-1} with various rotation speeds (400, 625, 900, 1225, 1600 and 2025 rpm), and the kinetic current density could be calculated from Koutecky-Levich plots using the following equation:

$$\frac{1}{j} = \frac{1}{j_k} + \frac{1}{j_D} \qquad \text{(S1)}$$

where j, j_k and j_D are the measuring current density, kinetic and diffusion limiting current density, respectively.

The rotating ring disk electrode (RRDE) polarization curves were also collected at 1600 rpm and 5 mV s^{-1}, and the potential of ring electrode was held at 1.3 V (vs. RHE). The electron transfer number (n) and the yields of peroxide (H_2O_2%) can be calculated by the following equations:

$$n = \frac{4I_D}{I_D + I_R/N} \tag{S2}$$

$$H_2O_2\% = 200\frac{I_R/N}{I_D + I_R/N} \tag{S3}$$

where I_D, I_R and N stand for the disk current, the ring current and current collection efficiency (Pt ring), respectively. In this measurement, N=0.4.

For the Tafel plot, the kinetic current density measured at a rate of 5 mV/s with a rotating speed from 1600 rpm was calculated from the mass-transport correction of the RDE data by:

$$J_K = \frac{J \times J_L}{(J_L - J)} \tag{S4}$$

The corresponding bifunctional activity parameter was evaluated by ΔE using the following equation:

$$\Delta E = E_{OER,j=10} - E_{ORR,j=-3} \tag{S5}$$

where $E_{OER,\ j=10}$ and $E_{ORR,\ j=-3}$ are the potential at 10 mA cm^{-2} for OER and -3 mA cm^{-2} for ORR, respectively.

The ORR stabilities were examined by chronoamperometry method at constant potential of -0.3 V (vs. SCE) for 10000 s. Furthermore, the poisoning durabilities were performed by the addition of CO and methanol. For comparison, the commercial 20% Pt/C and 10% Pt/C catalysts were operated with the same procedure.

5. Theoretical calculation details: Theoretical calculations have been performed within the framework of density functional theory (DFT) as implemented by the Vienna an initio Simulation Package (VASP).[2] The exchange-correlation energy was treated in the generalized-gradient approximation (GGA) using Perdew-Burke-Ernzerhof PBE method.[3] The nanotube model was constructed on the *x* direction of NCNT ($C_{70}N_2$), FeCo@NCNT

($C_{70}N_2$@$FeCo_3$) and NP-FeCo@NCNT ($C_{204}N_3P$@$FeCo_3$) with a 16 Å vacuum layer in the *ab* plane. The dipole correction was not used in the calculation. The cutoff energy of plane wave was chosen at 400 eV. For the structure optimizations, 1×1×6 Monkhorst-Pack (MP) grids were used. The changes in total energies between two successive iteration steps were less than 10^{-5} eV, and all the Hellmann-Feynman force acting on each atom was lower than 0.01 eV /Å. The adsorption free energies of O, *OH and* OOH on all structures were calculated by the formula $\Delta G = \Delta E + \Delta ZPE - T\Delta S$, where ΔE, ΔZPE, ΔS are the binding energy, zero point energy change and entropy change of H adsorption reaction, respectively. Herein, a solvation correction with energy equals to -0.22 eV is applied to only ΔE_{*OH} and ΔE_{*OOH} since water molecule could solvate *OH and *OOH moieties with hydrogen bond, whereas the hydrogen bond is absent for *O. For ORR and OER intermediates, the adsorption Gibbs free energies can be expressed by Ref.[4]

6. Fabrication of Rechargeable ZAB and Solid-state Zn-air battery: The performance of liquid Zn-air battery were inspected using electrochemical working station (Princeton Applied Research, P2000, USA) under ambient conditions. The rechargeable home-made Zn-air battery was assembled. The as-prepared FeCo@NCNT, NPC/FeCo@NCNT catalysts, and the commercial physically mixed catalysts of 20% Pt/C and RuO_2 (denoted as 20% Pt/C + RuO_2, mass ratio of 1:1) coated on gas diffusion layer based carbon paper were used as the air cathodes, and the geometric areas were ~1.0 cm^2, with the catalyst mass loading of 1.0 mg cm^{-2}. A polished Zn foil and 6 M KOH+0.2 M $Zn(Ac)_2$ mixture solution was employed as the anode and electrolyte, respectively.

For the assembling and evaluation of all-solid-state Zn-air battery, the air cathode was firstly fabricated by dropping the NPC/FeCo@NCNT ink on the carbon cloth, with mass loading of 1.0 mg cm^{-2}. The polished zinc plate was used as anode. The PVA-alkaline gel electrolyte was prepared as follows: 1 g polyvinyl alcohol powder was added in 10 mL DI water and stirred at ~90 °C until it was totally dissolved, then 1 mL of 18 M KOH + 0.2 M

Zinc acetate mixture solution was added and kept at ~90 ºC for ~1 h. Subsequently, the obtained solution was feezed at -10 ºC, and then thawed to obtain the gelatin for future use. Finally, the all-solid-state ZAB was assembled by pasting the as-prepared air electrode and polished Zn foil onto the two sides of PVA gel.

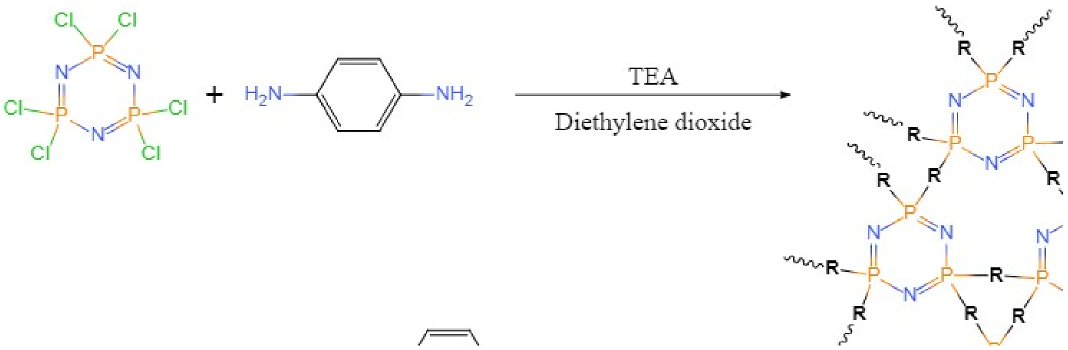

Figure S1. Schematic illustration of the preparation of polyphosphazenes.

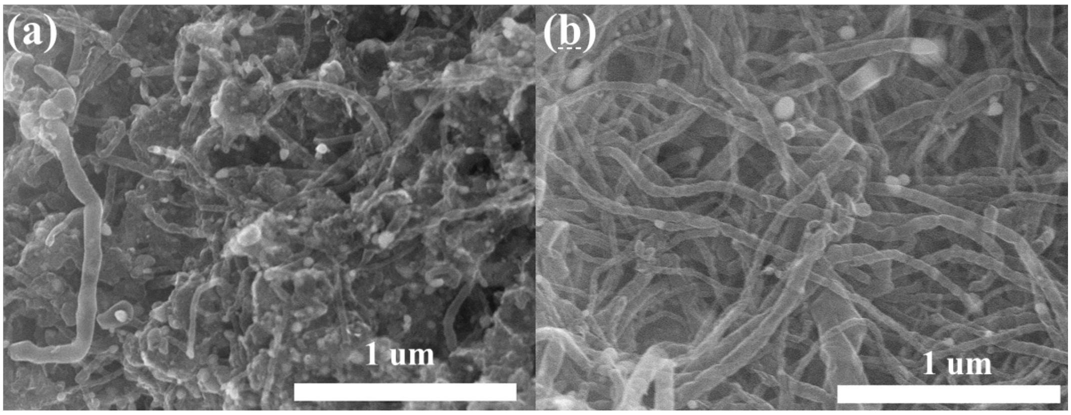

Figure S2. SEM images of FeCo@NCNT synthesized with (a) the low and (b) high concentrations of Fe^{3+} and Co^{2+}.

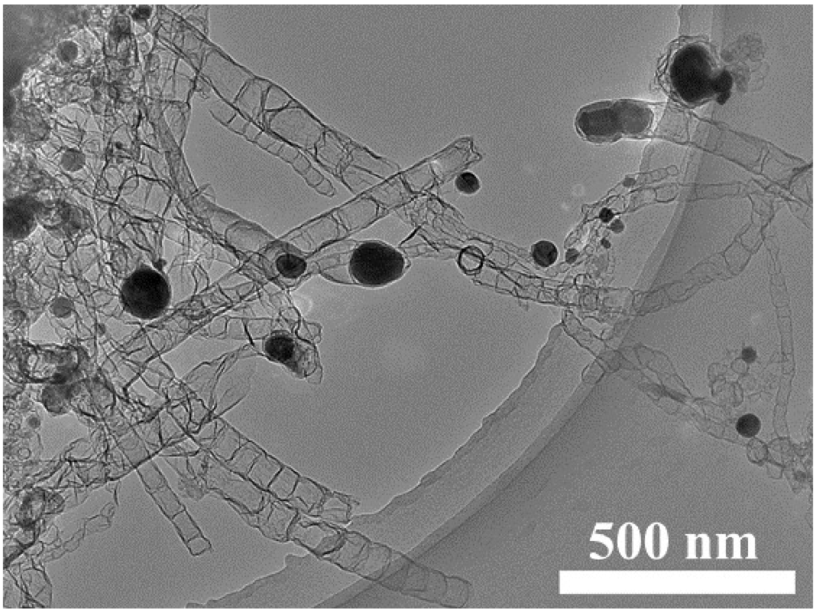

Figure S3. TEM image of FeCo@NCNT synthesized with the high concentrations of Fe^{3+} and Co^{2+}.

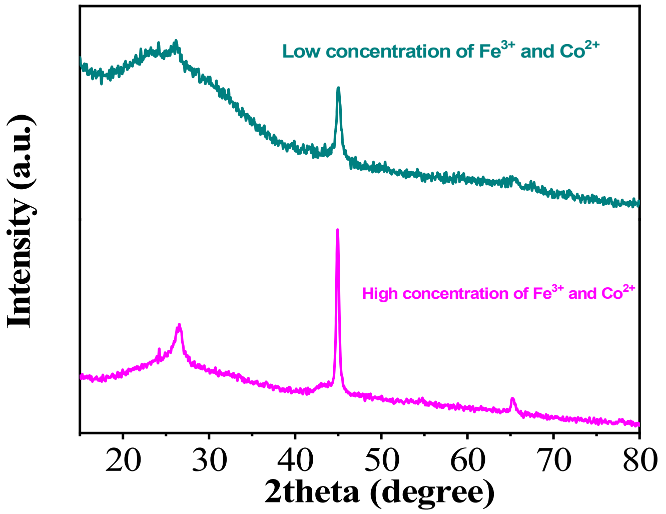

Figure S4. XRD patterns of FeCo@NCNT-short synthesized with (a) the low and (b) high concentrations of Fe^{3+} and Co^{2+}).

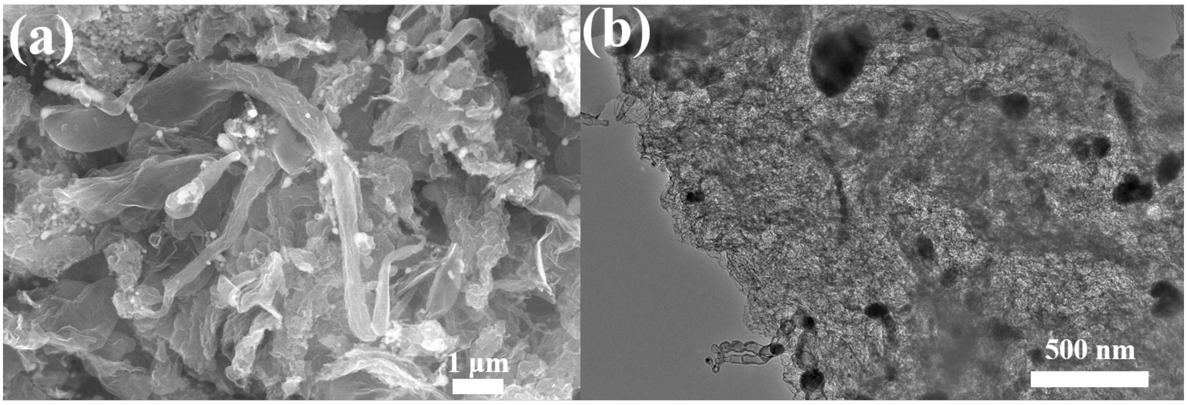

Figure S5. (a) SEM and (b) TEM images of FeCo@NPG synthesized without PSs template.

Table S1. Summary of element contents in the as-preapred samples determined by XPS.

	C	N	P	Fe	Co	O
**NHGS**	79.71	15.60	-	-	-	4.69
**FeCo@NCNT**	93.18	2.71	-	0.25	0.27	3.60
**NPC/FeCo@NCNT-thin**	90.73	3.29	0.78	0.23	0.24	4.73
**NPC/FeCo@NCNT**	84.22	4.80	2.77	0.21	0.21	7.79
**NPC/FeCo@NCNT-thick**	83.45	5.07	3.3	0.18	0.17	7.79
**NPC**	83.52	6.75	3.18	-	-	6.55

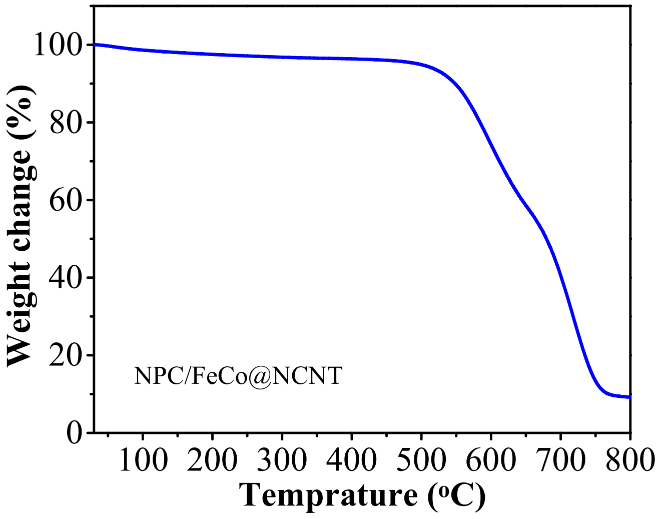

Figure S6. TG curves of NPC/FeCo@NCNT.

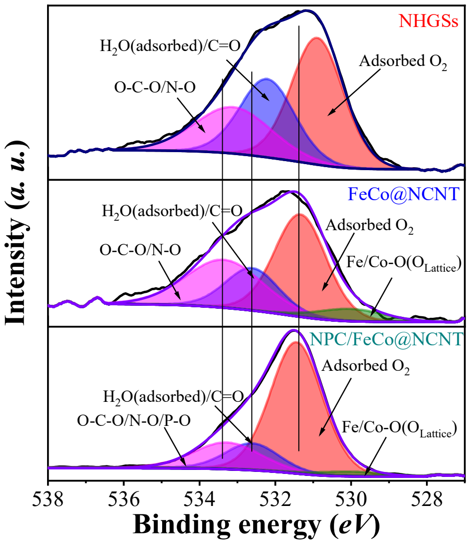

Figure S7. O 1s spectra of NHGSs, FeCo@NCNT, NPC/FeCo@NCNT.

Table S2. Oxygen species in the as-prepared samples determined by XPS using O 1s.

Peak	Assignment	Binding energy (eV)/fraction of species (%)		
		NHGSs	FeCo@NCNT	NPC/FeCo@NCNT
O1s	Fe/Co-O ($O_{Lattice}$)	-/-	530.0/6.58	530.0/2.68
	Adsorbed O_2	530.9/45.66	531.3/44.26	531.3/66.52
	H_2O(adsorbed)/C=O	532.2/29.66	532.6/18.67	532.6/13.69
	O-C-O/N-O	533.1/24.68	533.3/30.49	533.3/17.12

Table S3. Carbon species in the as-prepared samples determined by XPS using C 1s.

	C-C		C-O&C=N&C-P		C=O&C-N		O-C=O	
	Binding energy (eV)	Content (%)	Binding energy (eV)	Content (%)	Binding energy (eV)	Content (%)	Binding energy (eV)	Content (%)
**NHGS**	284.59	43.22	285.55	28.80	286.81	14.21	289.26	13.77
**FeCo@NCNT**	284.78	50.21	285.5	23.25	286.8	11.85	289.64	14.69
**NPC/FeCo@NCNT-thin**	284.75	61.28	285.6	15.62	286.76	10.09	290.75	13.00
**NPC/FeCo@NCNT**	284.77	53.23	285.6	20.00	286.8	10.22	290.29	16.54
**NPC/FeCo@NCNT-thick**	284.77	58.82	285.6	16.28	286.54	10.35	289.42	14.55
**NPC**	284.69	56.30	285.65	17.15	286.61	12.65	289.43	13.90

Table S4. Nitrogen species in the as-prepared samples determined by XPS using N 1s.

	Pyridinic-N		Pyrrolic-N		Graphitic-N		Oxidized-N	
	Binding energy (eV)	Content (%)	Binding energy (eV)	Content (%)	Binding energy (eV)	Content (%)	Binding energy (eV)	Content (%)
**NHGS**	398.19	39.09	399.42	16.61	400.70	34.60	403.09	9.71
**FeCo@NCNT**	398.50	26.68	399.76	17.53	401.09	40.45	403.02	15.33
**NPC/FeCo@NCNT-thin**	398.74	42.25	399.85	2.03	401.12	44.20	403.00	11.53
**NPC/FeCo@NCNT**	398.77	42.74	399.94	7.31	401.16	37.96	403.51	12.00
**NPC/FeCo@NCNT-thick**	398.78	38.60	400.15	6.24	401.20	48.28	403.34	6.88
**NPC**	398.19	23.03	399.47	10.59	400.83	58.10	403.29	8.28

Table S5. Phosphorus species in the as-preapred samples determined by XPS using P 2p.

	P-C		P-O	
	Binding energy (eV)	Content (%)	Binding energy (eV)	Content (%)
**NPC**	133.04	77.44	133.92	22.56
**NPC/FeCo@NCNT-thin**	133.48	79.56	134.38	20.43
**NPC/FeCo@NCNT**	133.65	80.77	134.72	19.22
**NPC/FeCo@NCNT-thick**	133.81	78.47	134.77	21.53

Table S6. Comparison of OER/ORR electrocatalytic properties of the NPC/FeCo@NCNT catalyst with recently reported advanced catalysts.

**References**	**Catalysts**	**OER: E_{j10} (mV)**	**ORR** (0.1 M KOH)			**Δ E (V)**
			**E_{onset} (V vs. RHE)**	**$E_{1/2}$ (V vs. RHE)**	**Limiting current density (mA cm^{-2})**	
**This work**	**NPC/FeCo@NCNT**	**339 (0.1 M KOH)**	**0.87**	**0.835**	**5.7**	**0.741**
[5]	FCx-NC/CNTs-10	360 (0.1 M KOH)	0.90	0.79	NG	0.8
[6]	FeCo/FeCoNi @NCNTs-HF	378 (0.1 M KOH)	NG	0.85	5.775	0.758

[7]	FeNi-NC	380 (0.1 M KOH)	0.98	0.83	4.84	0.81
[8]	CoDNi-N/C	360 (0.1 M KOH)	0.92	0.81	6.7	0.78
[9]	FeNx/PNC	395 (0.1 M KOH)	0.997	0.86	5.95	0.775
[10]	FeCo/N-DNC	390 (0.1 M KOH)	0.89	0.81	NG	0.81
[11]	CoFe/N-GCT	500 (0.1 M KOH)	0.91	0.79	NG	0.88
[4]	N-GCNT/FeCo-3	500 (0.1 M KOH)	1.03	0.92	5.4	0.81
[12]	NiCo/NLG-270	340 (0.1 M KOH)	NG	0.82	NG	0.75
[13]	meso/micro-FeCo-Nx-CN)	480 (1 M KOH)	0.954	0.886	6.3	0.878

NG: not given

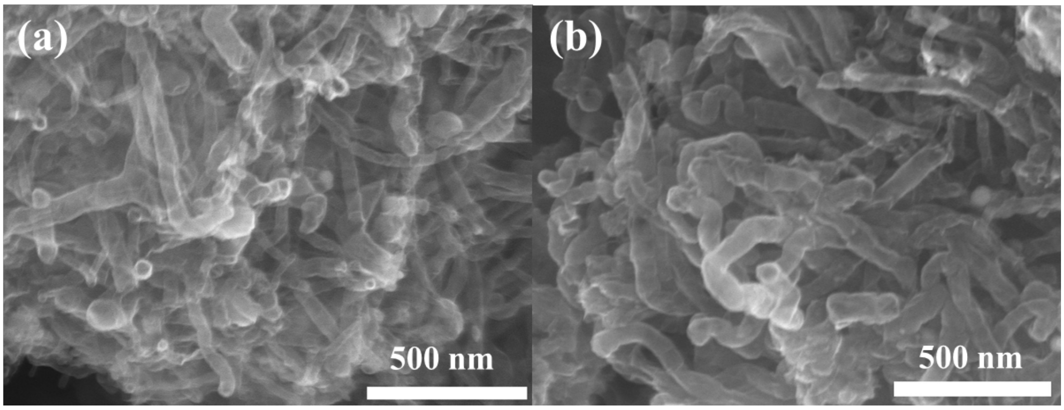

Figure S8. SEM images of NPC/FeCo@NCNT-thin (a) and NPC/FeCo@NCNT-thick (b).

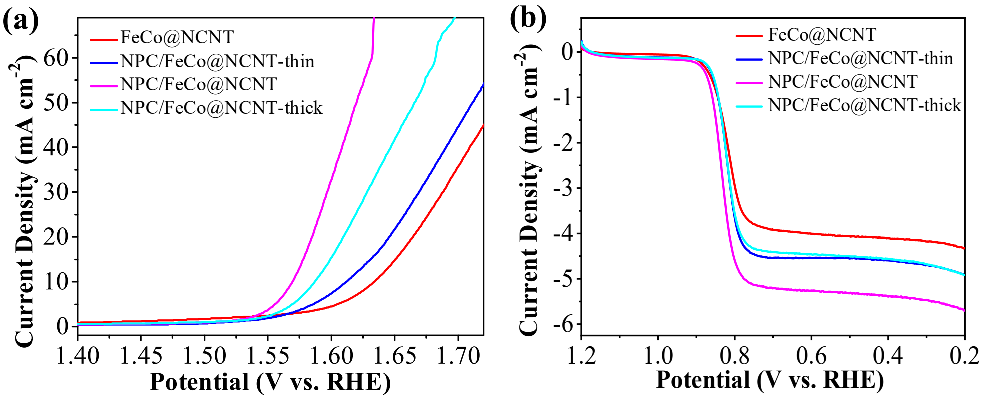

Figure S9. OER (a) and ORR (b) performances of FeCo@NCNT, NPC/FeCo@NCNT-thin, NPC/FeCo@NCNT and NPC/FeCo@NCNT-thick.

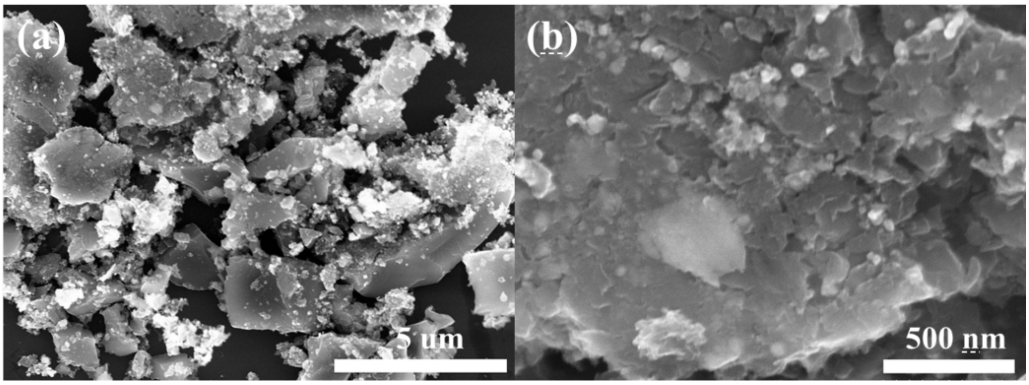

Figure S10. SEM images of NPC sample.

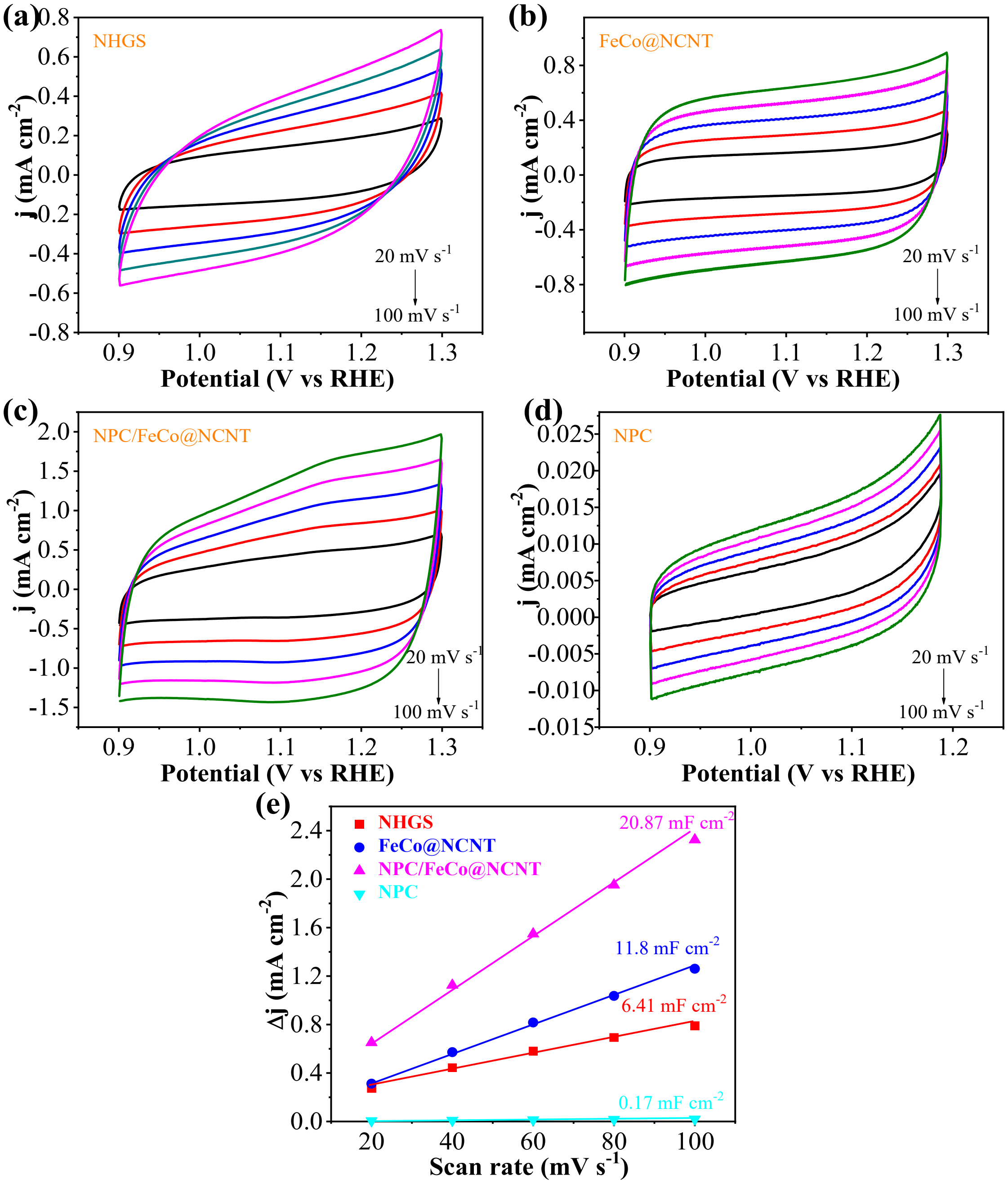

Figure S11. CV curves of NHGS (a), FeCo@NCNT (b), NPC/FeCo@NCNT (c) and NPC (d) in O_2-saturated 0.1 M KOH at different scan rates; (e) ECSA.

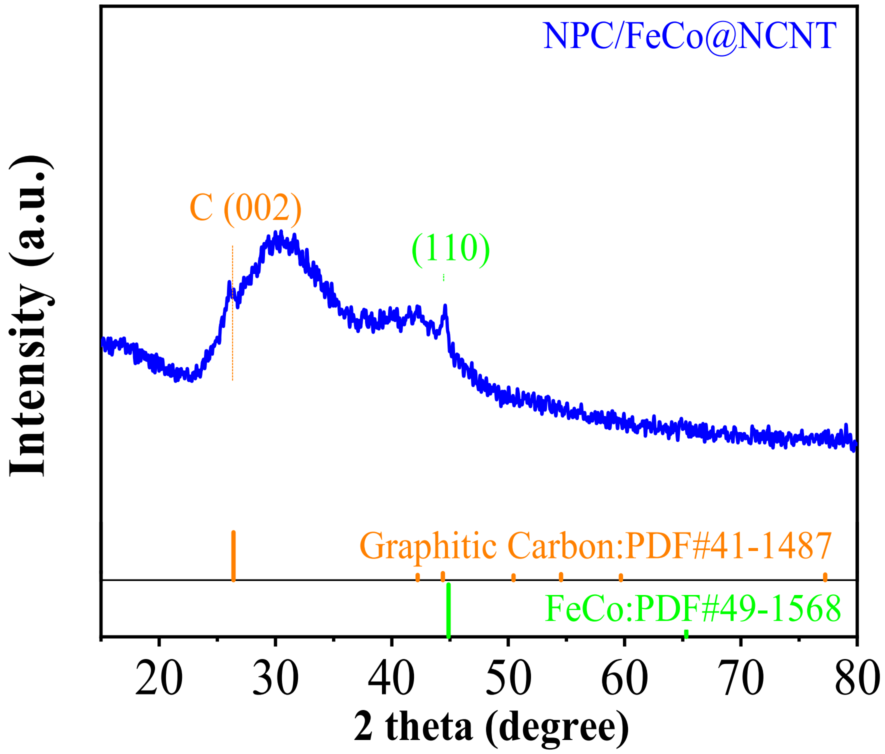

Figure S12. XRD pattern of NPC/FeCo@NCNT after OER stabilities test.

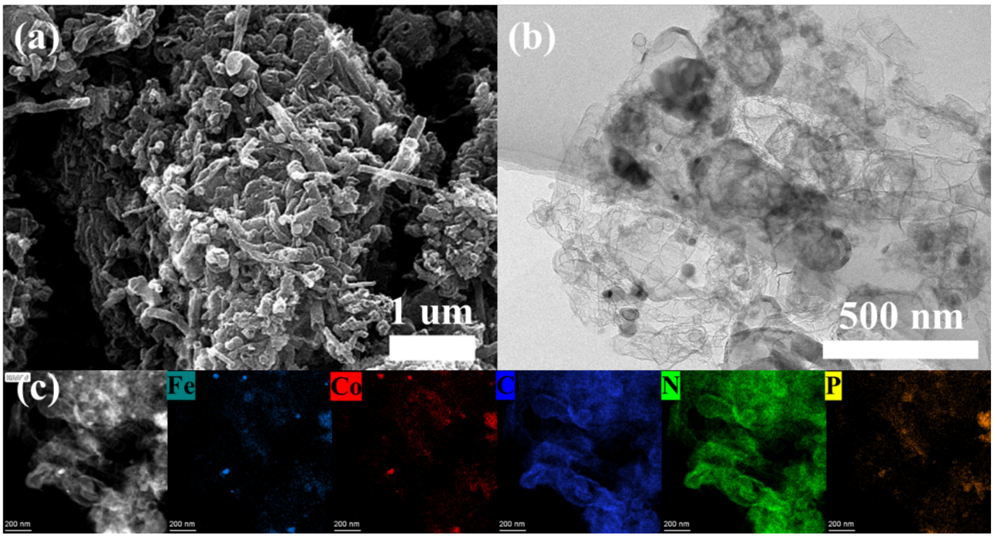

Figure S13. SEM images (a), TEM (b) and element mapping images (c) of NPC/FeCo@NCNT after OER stability test.

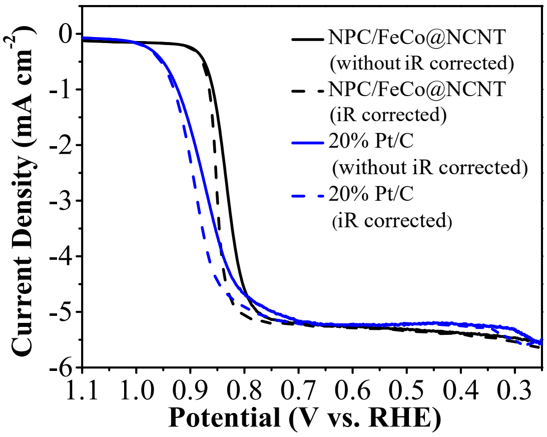

Figure S14. ORR polarization curves comparison before and after iR compensation in 0.1 M KOH (R≈35 Ω).

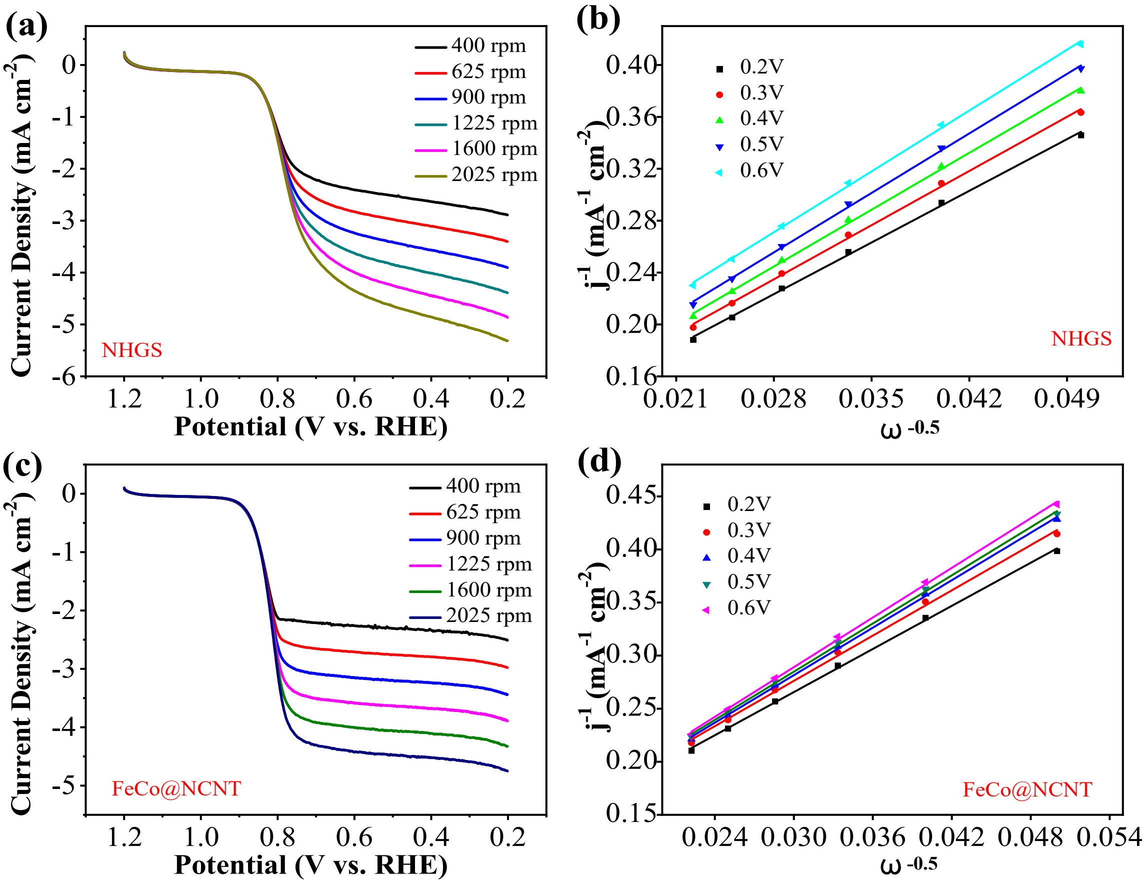

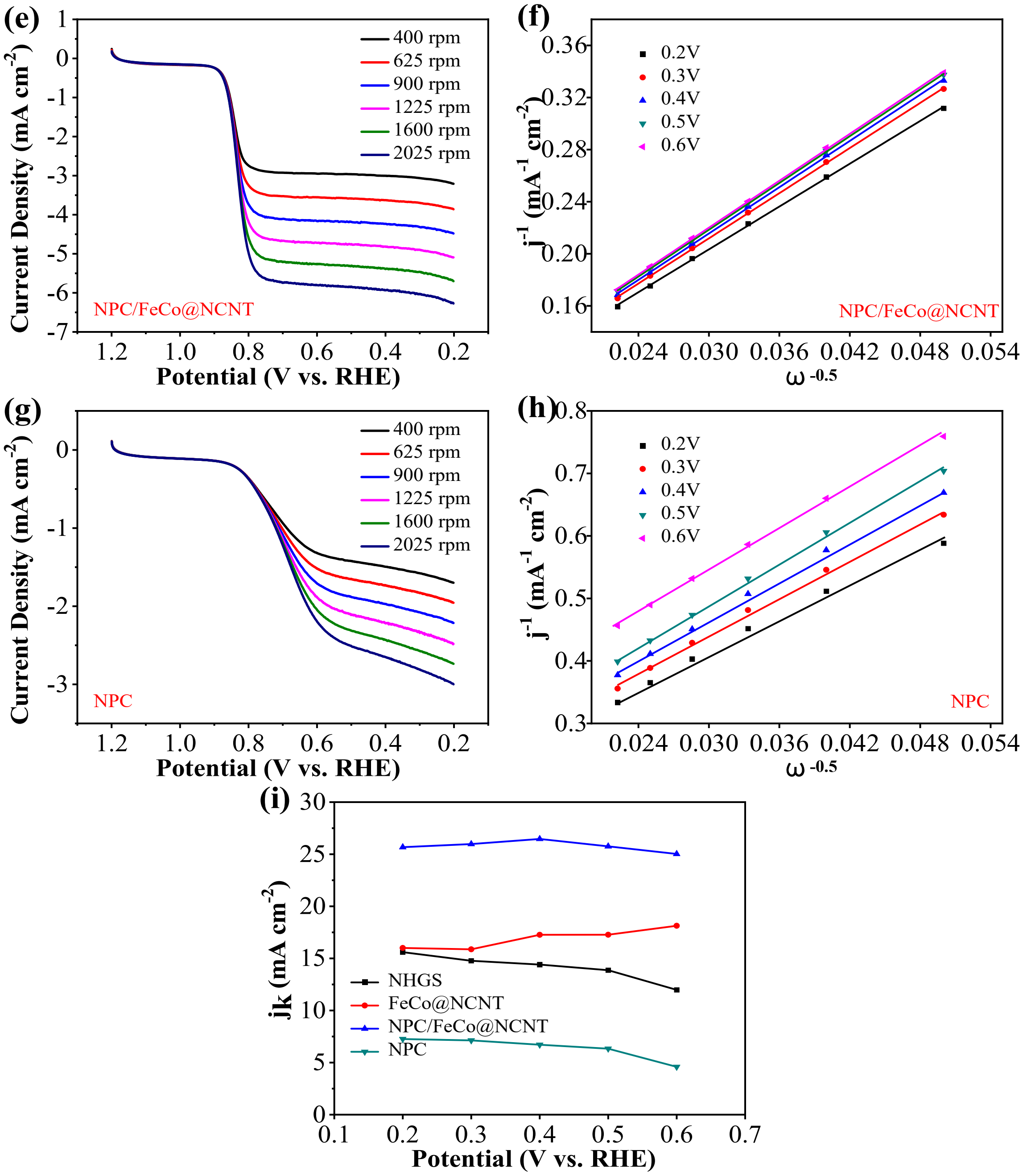

Figure S15. Rotating disk electrode LSVs of NHGS (a), FeCo@NCNT (c), NPC/FeCo@NCNT (e) and NPC (g) in O_2-saturated 0.1 M KOH at different rotation rates (scan rate: 5 mV s^{-1}); Koutecky-Levich plots of ORR obtained at NHGS (b), FeCo@NCNT (d), NPC/FeCo@NCNT (f) and NPC (h) based on the data extracted from (a, c, e, g); (i) kinetic current densities.

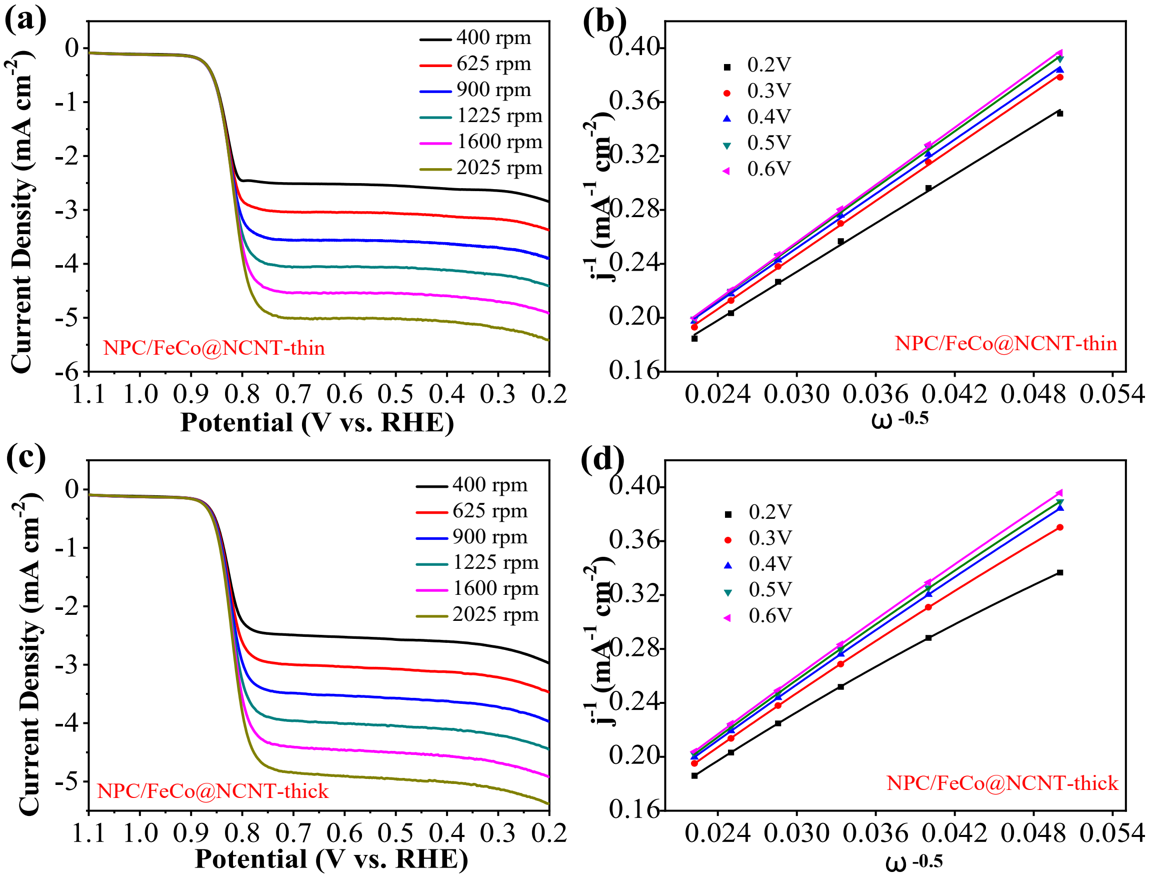

Figure S16. Rotating disk electrode LSVs of NPC/FeCo@NCNT-thin (a) and NPC/FeCo@NCNT-thick (c) in O_2-saturated 0.1 M KOH at different rotation rates (scan rate: 5 mV s^{-1}); Koutecky-Levich plots of ORR obtained at NPC/FeCo@NCNT-thin (b) and NPC/FeCo@NCNT-thick (d).

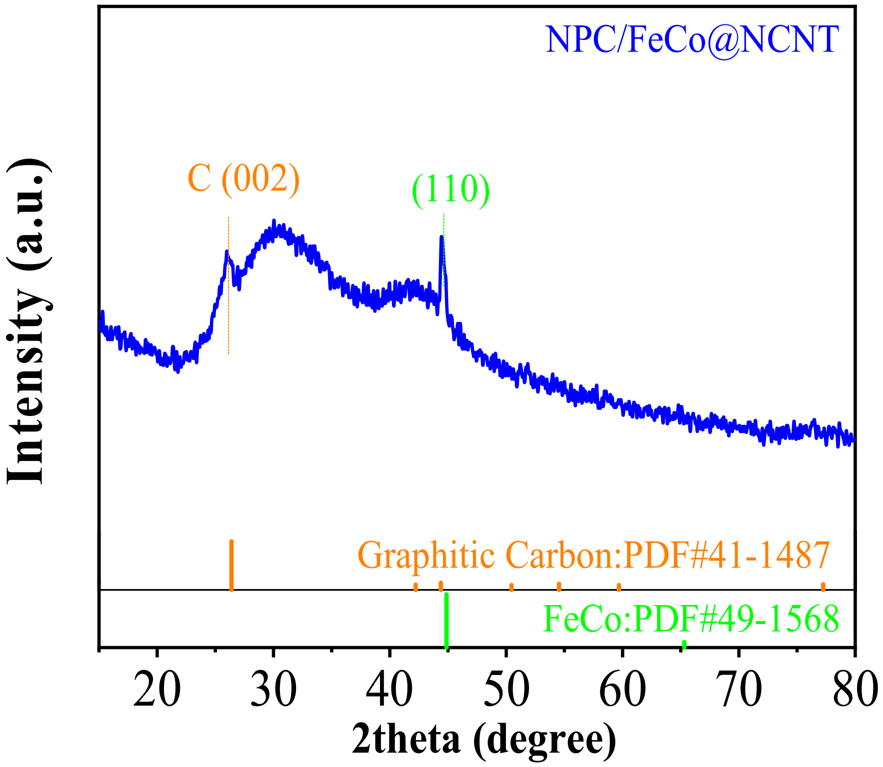

Figure S17. XRD pattern of NPC/FeCo@NCNT after ORR stabilities test.

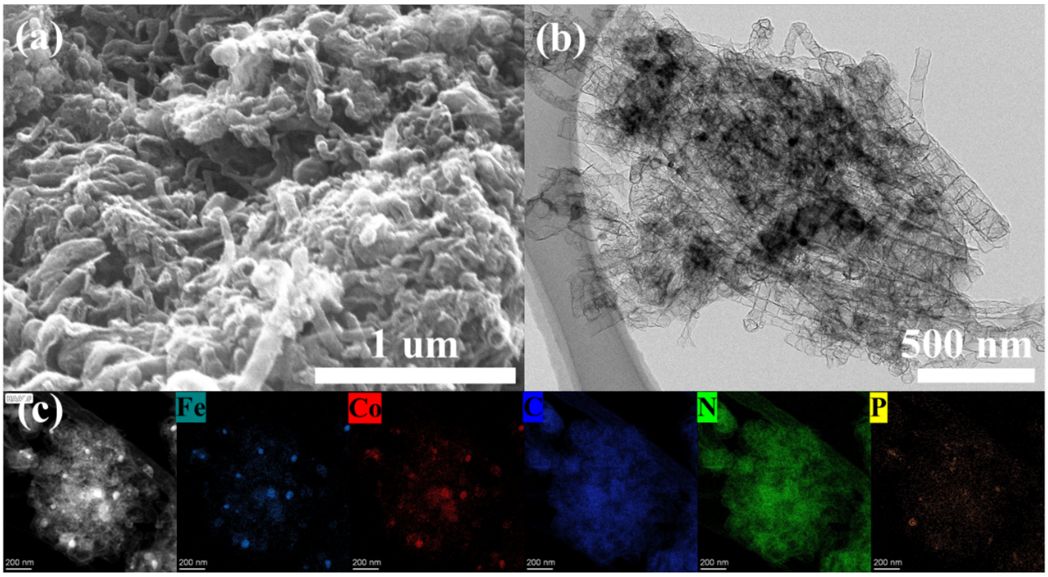

Figure S18. SEM images (a), TEM (b) and element mapping images (c) of NPC/FeCo@NCNT after ORR stability test.

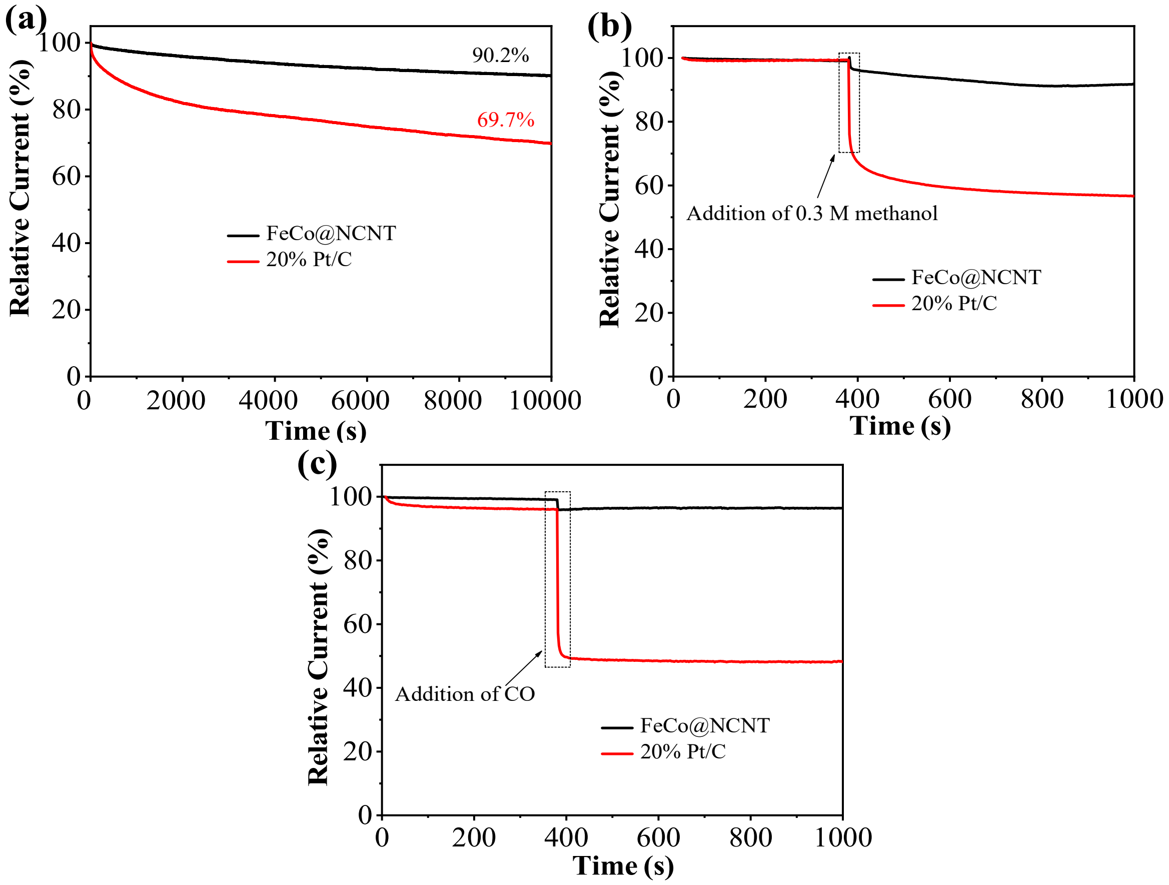

Figure S19. Stability evaluation of FeCo@NCNT(a); (b-c) Durability evaluation, the arrows indicate the addition of methanol and CO, respectively.

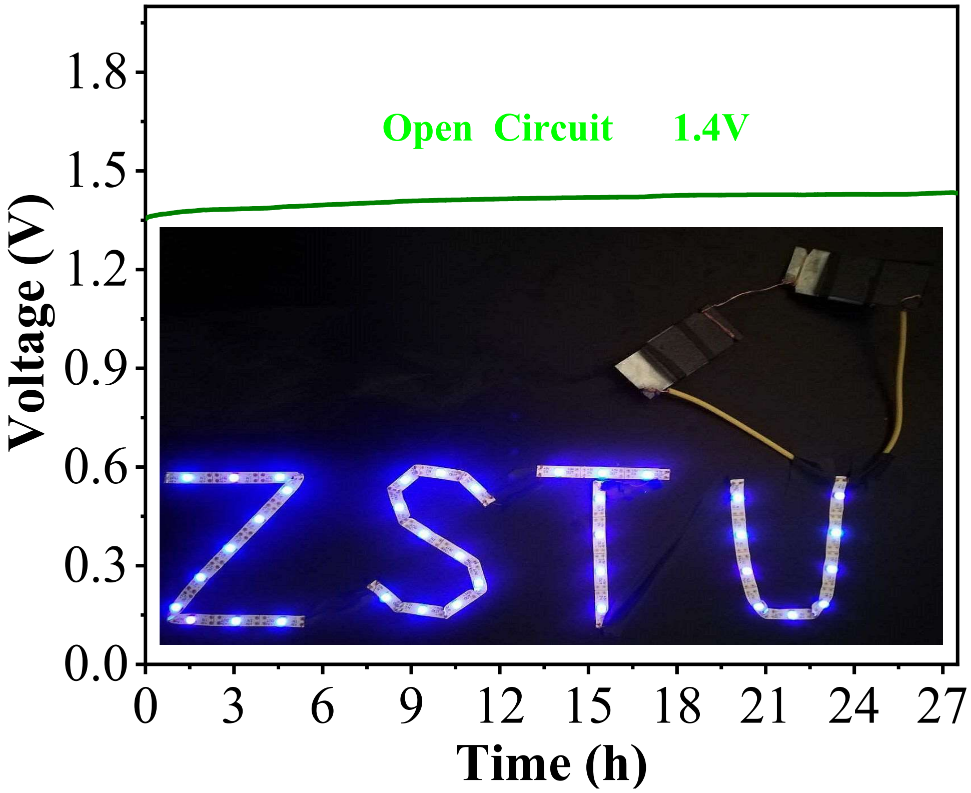

Figure S20. Open circuit plot (inset: a photograph of the ZSTU working-LED lighted by two Zn-air batteries with 2 mg FeCo@NCNT).

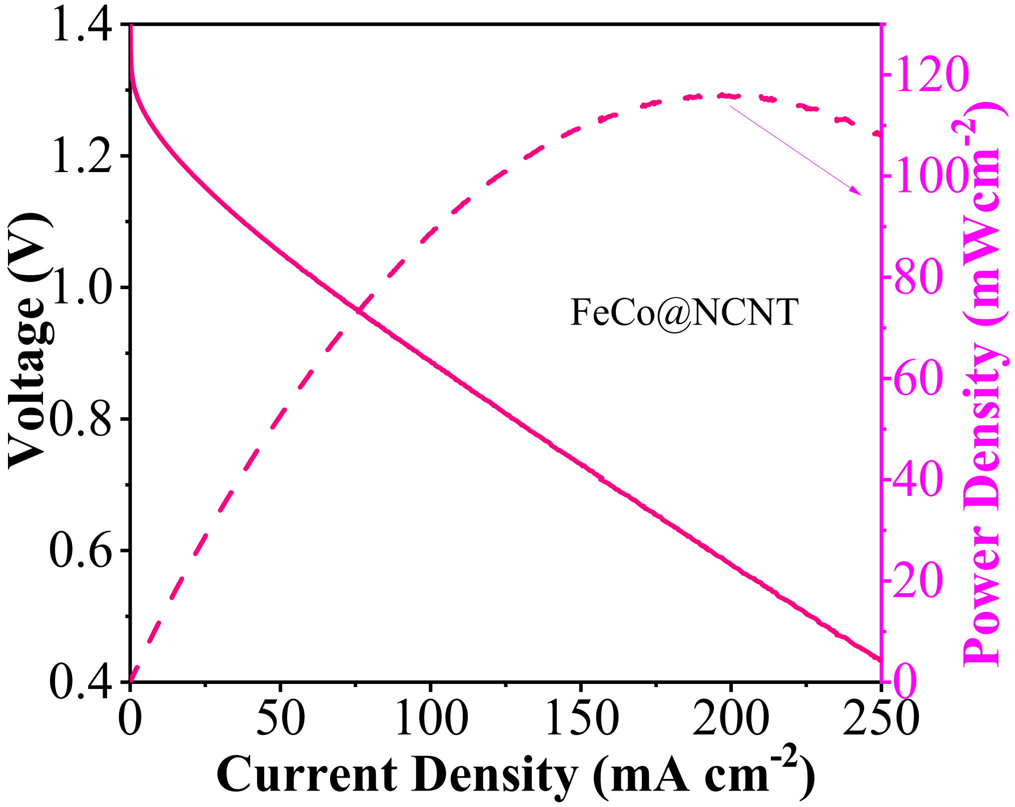

Figure S21. Polarization curves and power density curves of Zn-air batteries based with FeCo@NCNT.

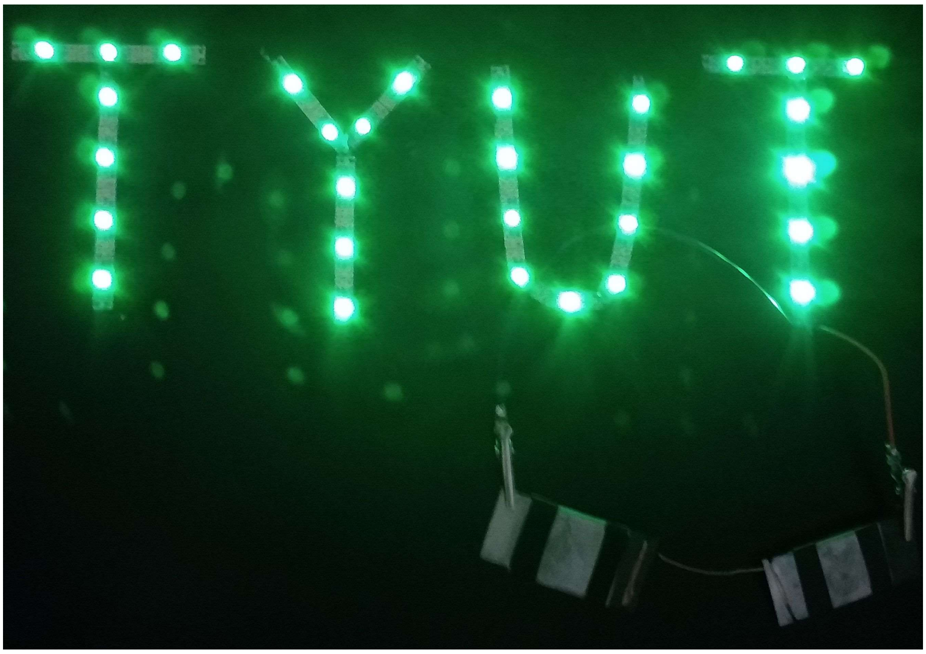

Figure S22. A photograph of the TYUT working-LED lighted by two Zn-air batteries with 2 mg NPC/FeCo@NCNT).

Table S7. Comparison of liquid and solid Zn-air performances of the NP-Fe_2Co_2@NCNT(1) catalyst with recently reported advanced catalysts.

References	Catalysts	liquid Zn-air battery		solid Zn-air battery	
		power density (mW cm^{-2})	specific capacity (mA h g^{-1})	open-circuit voltage (V)	power density (mW cm^{-2})
**This work**	**NPC/FeCo@ NCNT**	**151.3**	**810 (200 mA cm^{-2})**	**1.45**	**65.0**
[14]	NGM-Co	152	750 (20 mA cm^{-2})	1.439	～28
[15]	CoN_4/NG	-	730 (100 mA cm^{-2})	-	～28
[16]	CoSx/Co-NC-800	103	734.2 (40 mA cm^{-2})	1.34	-
[17]	o-CC-H2	91.4	707 (20 mA cm^{-2})	1.258	-
[18]	Fe-Co_4N@N-C	105	806 (5 mA cm^{-2})	1.34	72
[19]	Co/Co–N–C	132	-	1.41	-
[20]	(Zn,Co)/NSC	150	-	1.56	15
[21]	IOSHs-NSC	133	768 (10 mA cm^{-2})	1.408	60
[4]	N-GCNT/FeCo	89.3	653.2 (100 mA cm^{-2})	1.249	97.8

References

[1] X. Hao, Z. Jiang, X. Tian, X. Hao, Z.-J. Jiang, *Electrochim. Acta* **2017**, *253*, 21-30.

[2] P. E. Blöchl, *Phys. Rev. B* **1994**, *50*, 17953-17979.

[3] M. C. Payne, M. P. Teter, D. C. Allan, T. A. Arias, J. D. Joannopoulos, *Rev. Mod. Phys.* **1992**, *64*, 1045-1097.

[4] C.-Y. Su, H. Cheng, W. Li, Z.-Q. Liu, N. Li, Z. Hou, F.-Q. Bai, H.-X. Zhang, T.-Y. Ma, *Adv. Energy Mater.* **2017**, *7*, 1602420.

[5] X. Xiao, X. Li, G. Yu, J. Wang, G. Yan, Z. Wang, H. Guo, *J. Power Sources* **2019**, *438*, 227019.

[6] Z. Wang, J. Ang, B. Zhang, Y. Zhang, X. Y. D. Ma, T. Yan, J. Liu, B. Che, Y. Huang, X. Lu, *Appl. Catal. B-Environ.* **2019**, *254*, 26-36.

[7] L. Yang, X. Zeng, D. Wang, D. Cao, *Energy Stor. Mater.* **2018**, *12*, 277-283.

[8] Z. Li, H. He, H. Cao, S. Sun, W. Diao, D. Gao, P. Lu, S. Zhang, Z. Guo, M. Li, R. Liu, D. Ren, C. Liu, Y. Zhang, Z. Yang, J. Jiang, G. Zhang, *Appl. Catal. B-Environ.* **2019**, *240*, 112-121.

[9] L. Ma, S. Chen, Z. Pei, Y. Huang, G. Liang, F. Mo, Q. Yang, J. Su, Y. Gao, J. A. Zapien, C. Zhi, *ACS Nano* **2018**, *12*, 1949-1958.

[10] G. Fu, Y. Liu, Y. Chen, Y. Tang, J. B. Goodenough, J.-M. Lee, *Nanoscale* **2018**, *10*, 19937-19944.

[11] X. Liu, L. Wang, P. Yu, C. Tian, F. Sun, J. Ma, W. Li, H. Fu, *Angew. Chem. Int. Edit.* **2018**, *130*, 16398-16402.

[12] X.-R. Wang, J.-Y. Liu, Z.-W. Liu, W.-C. Wang, J. Luo, X.-P. Han, X.-W. Du, S.-Z. Qiao, J. Yang, *Adv. Mater.* **2018**, *30*, 1800005.

[13] S. Li, C. Cheng, X. Zhao, J. Schmidt, A. Thomas, *Angew. Chem. Int. Edit.* **2018**, *57*, 1856-1862.

[14] C. Tang, B. Wang, H.-F. Wang, Q. Zhang, *Adv. Mater.* **2017**, *29*, 1703185.

[15] L. Yang, L. Shi, D. Wang, Y. Lv, D. Cao, *Nano Energy* **2018**, *50*, 691-698.

[16] Q. Lu, J. Yu, X. Zou, K. Liao, P. Tan, W. Zhou, M. Ni, Z. Shao, *Adv. Funct. Mater.* **2019**, *29*, 1904481.

[17] H.-F. Wang, C. Tang, B. Wang, B.-Q. Li, X. Cui, Q. Zhang, *Energy Stor. Mater.* **2018**, *15*, 124-130.

[18] Q. Xu, H. Jiang, Y. Li, D. Liang, Y. Hu, C. Li, *Appl. Catal. B-Environ.* **2019**, *256*, 117893.

[19] P. Yu, L. Wang, F. Sun, Y. Xie, X. Liu, J. Ma, X. Wang, C. Tian, J. Li, H. Fu, *Adv. Mater.* **2019**, *31*, 1901666.

[20] D. Liu, B. Wang, H. Li, S. Huang, M. Liu, J. Wang, Q. Wang, J. Zhang, Y. Zhao, *Nano Energy* **2019**, *58*, 277-283.

[21] K. Tang, C. Yuan, Y. Xiong, H. Hu, M. Wu, *Appl. Catal. B-Environ.* **2020**, *260*, 118209.
